# Safety, immunogenicity and protective effect of sequential vaccination with inactivated and recombinant protein COVID-19 vaccine in the elderly: a prospective longitudinal study

**DOI:** 10.1038/s41392-024-01846-9

**Published:** 2024-05-13

**Authors:** Hong-Hong Liu, Yunbo Xie, Bao-Peng Yang, Huan-Yue Wen, Peng-Hui Yang, Jin-E Lu, Yan Liu, Xi Chen, Meng-Meng Qu, Yang Zhang, Wei-Guo Hong, Yong-Gang Li, Junliang Fu, Fu-Sheng Wang

**Affiliations:** 1 Out-patient Department of Day Diagnosis and Treatment, The Fifth Medical Center of Chinese PLA General Hospital, Beijing, 100039 China; 2 Senior Department of Infectious Diseases, The Fifth Medical Center of Chinese PLA General Hospital, National Clinical Research Center for Infectious Diseases, Beijing, 100039 China; 3 Chinese PLA Medical School, Chinese PLA General Hospital, Beijing, 100039 China; 4 Hunyuan County People’s Hospital, Datong, 037499 Shanxi Province China; 5 Faculty of Hepato-Pancreato-Biliary Surgery, Institute of Hepatobiliary Surgery, The First Medical Center, Chinese PLA General Hospital, Beijing, 100853 China

**Keywords:** Clinical trials, Vaccines, Ageing, Outcomes research, Infectious diseases

## Abstract

The safety and efficacy of COVID-19 vaccines in the elderly, a high-risk group for severe COVID-19 infection, have not been fully understood. To clarify these issues, this prospective study followed up 157 elderly and 73 young participants for 16 months and compared the safety, immunogenicity, and efficacy of two doses of the inactivated vaccine BBIBP-CorV followed by a booster dose of the recombinant protein vaccine ZF2001. The results showed that this vaccination protocol was safe and tolerable in the elderly. After administering two doses of the BBIBP-CorV, the positivity rates and titers of neutralizing and anti-RBD antibodies in the elderly were significantly lower than those in the young individuals. After the ZF2001 booster dose, the antibody-positive rates in the elderly were comparable to those in the young; however, the antibody titers remained lower. Gender, age, and underlying diseases were independently associated with vaccine immunogenicity in elderly individuals. The pseudovirus neutralization assay showed that, compared with those after receiving two doses of BBIBP-CorV priming, some participants obtained immunological protection against BA.5 and BF.7 after receiving the ZF2001 booster. Breakthrough infection symptoms last longer in the infected elderly and pre-infection antibody titers were negatively associated with the severity of post-infection symptoms. The antibody levels in the elderly increased significantly after breakthrough infection but were still lower than those in the young. Our data suggest that multiple booster vaccinations at short intervals to maintain high antibody levels may be an effective strategy for protecting the elderly against COVID-19.

## Introduction

The coronavirus disease 2019 (COVID-19) pandemic, originating in late 2019, has affected over 670 million individuals globally, resulting in more than 6 million fatalities. These developments have had a significant influence on the world economy and healthcare infrastructure. Despite the World Health Organization (WHO) declaring on May 5, 2023 that the COVID-19 outbreak no longer meets the criteria of a public health emergency of international concern, it emphasized that this does not mean that COVID-19 no longer poses a global health risk.^1^ With the emergence of new variants and frequent instances of breakthrough infections, the repercussions of COVID-19 might persist longer than anticipated, leading countries to maintain their alertness in combating it.

Since the elderly have lower resistance to the virus, the COVID-19 pandemic has posed unparalleled health risks to this demographic group, especially those with underlying diseases, such as hypertension, diabetes, cardiovascular disease, and chronic respiratory disease.^2,3^ Research indicates that elderly individuals have higher levels of angiotensin-converting enzyme 2 (ACE2) compared to younger adults,^4^ which elevates their vulnerability to SARS-CoV-2 infection. Outbreak data from countries such as China, Italy, Japan, Singapore, Canada, and South Korea illustrate that the elderly are more susceptible to COVID-19.^5^ A meta-analysis of 59 studies involving 36,470 participants revealed that people over the age of 70 had a 65% higher risk of contracting COVID-19 than those under the age of 70.^6^ Similarly, a study on the epidemiology of SARS-CoV-2 in China reported a notable susceptibility to COVID-19 among individuals aged over 60 compared to younger and middle-aged adults.^7^ Additionally, the immune system of elderly individuals is often in a chronic, prolonged, pro-inflammatory state associated with the aging process. Continuously low levels of innate immune response and heightened levels of pro-inflammatory cytokines could worsen infection-induced tissue damage and advance the progression of COVID-19.^8^ Singhal et al. reported that COVID-19 patients of advanced age exhibited a severity rate of ~50% and a mortality rate of 10%.^9^

Vaccines are a key means of epidemic prevention and control. Since the onset of COVID-19, global vaccine research has entered an arms race. Thus far, of the 199 vaccines undergoing preclinical research, 176 have progressed to the clinical research stage. As of March 2023, China has approved the large-scale use of 14 vaccines across five categories: inactivated vaccines, recombinant protein vaccines, live attenuated influenza virus vector vaccines, mRNA vaccines, and adenovirus vector vaccines. The protective effect of the COVID-19 vaccine is closely related to the production of neutralizing antibodies, the establishment of immune memory, and the production of virus-specific T cells. Vaccination to prevent illness and reduce the number of severe cases and deaths is an important measure to ensure the health of the elderly population. Related data indicate that unvaccinated individuals face a fivefold higher risk of SARS-CoV-2 infection compared to those who have received established vaccines.^10^ Additionally, the risk of hospitalization and mortality increases by more than 10 times for the unvaccinated group.^10^ The incidence rate ratio is directly related to vaccine efficacy.^10^ Overall, the consistent efficacy of the vaccine against severe COVID-19 remains exceptionally high.^10^ Currently, the evolutionary trajectory of SARS-CoV-2 is becoming more focused, with the majority of recent variants stemming from the subvariants of the Omicron lineage.^11^ This trend indicates positive prospects for the development of novel vaccines. However, there is growing concern about the spread of these subvariants. While maintaining a binding affinity to ACE2 similar to the original strain, the additional mutation of the spike protein in these subvariants makes them prone to evading antibodies.^11^ This diminishes the efficacy of neutralizing antibodies, potentially leading to the uncontrolled spread of recent variants among vulnerable populations. The provision of optimal immunization strategies to maximize the immunogenicity of existing vaccines in the context of the ongoing variability of COVID-19 remains to be further explored.

While investigations into the safety and effectiveness of COVID-19 vaccination in populations requiring additional safeguarding, such as cancer and AIDS patients,^12,13^ have commenced, research relevant to the elderly remains in its nascent stages. According to a recent study conducted in China, the COVID-19 vaccine inoculation rate among the elderly, especially those aged over 70 years, is low.^14^ This vaccination hesitancy is believed to be driven by concerns about contraindications and side effects and may have contributed to excessive mortality during the COVID-19 pandemic.^14^ Although several clinical studies have focused on the immune responses of the elderly to COVID-19 vaccines,^15,16^ there remains a dearth of convincing evidence regarding the safety and immunogenicity of the vaccines in this population—especially due to a lack of prospective, real-world, long-term follow-up clinical studies. It is also unclear whether the humoral immune response and protective effects induced by the COVID-19 vaccine in the elderly differ from those in the young population. To answer these questions, we conducted a prospective clinical observational study over 16 months to assess the safety and immunogenicity of two doses of an inactivated vaccine, BBIBP-CorV, followed by a recombinant protein vaccine booster, ZF2001, in elderly individuals.

## Results

### Baseline characteristics of participants

**Fig. 1:**
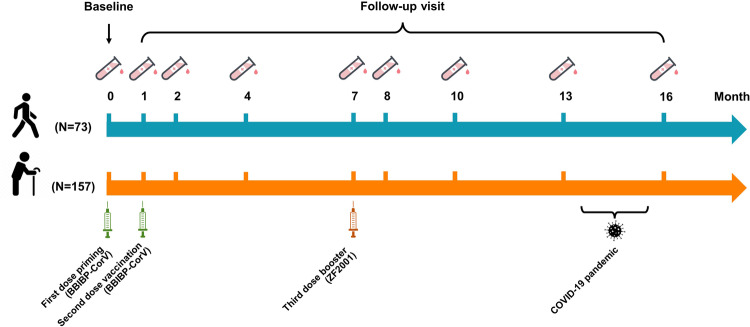
Study design. The first two doses of inactivated vaccine BBIBP-CorV were inoculated intramuscularly at 1-month intervals (actually 21–28 days) and the third dose of heterologous recombinant protein booster ZF2001 was administered 6 months after the second dose. The COVID-19 pandemic occurred between the 13th and 16th month. Blood samples were collected at baseline and the 1st, 2nd, 4th, 7th, 8th, 10th, 13th, and 16th months

As shown in Fig. 1 and Supplementary Table 1, from July 29, 2021, to August 3, 2021, 230 participants were enrolled in the vaccination and follow-up visit protocol. The baseline characteristics of all participants are listed in Table 1. Sex and body mass index (BMI) status did not differ significantly between the two groups. The elderly group’s blood glucose (6.05 vs 5.33, *P* < 0.0001), blood urine nitrogen (6.12 vs 5.27, *P* < 0.0001), and creatinine (70.53 vs 65.49, *P* = 0.001) levels were marginally higher than those of the young group. Elderly individuals were more likely to have underlying diseases (68.15% vs 19.18%, *P* < 0.0001). The prevalence of type 2 diabetes and hypertension (15.92% vs 4.11%, *P* = 0.011 and 20.38% vs 4.11%, *P* = 0.001, respectively) was significantly higher in the elderly.

**Table 1:** Baseline characteristics of the participants

	Young group (*n* = 73)	Elderly group (*n* = 157)	*p*
Age			
Years	48 (26–59)	69 (60–80)	**<0.0001**
Sex			0.727
Female	39 (53.42%)	80 (50.96%)	
Male	34 (46.58%)	77 (49.04%)	
BMI			0.694
<25 (kg/m^2^)	45 (61.64%)	101 (64.33%)	
≥25 (kg/m^2^)	28 (38.36%)	56 (35.67%)	
Laboratory tests^a^			
ALT (U/L)	20.46 (9.66–83.68)	20.99 (5.00–91.85)	0.673
AST (U/L)	22.89 (12.23–57.31)	23.60 (6.02–116.40)	0.163
TBIL (µmol/L)	15.20 (6.21–66.05)	14.37 (5.11–36.86)	0.550
TC (mmol/L)	4.60 (1.86–6.86)	4.90 (1.70–7.61)	0.075
TG (mmol/L)	1.42 (0.44–11.94)	1.54 (0.38–8.87)	0.373
GLU (mmol/L)	5.33 (1.97–20.51)	6.05 (3.47–25.72)	**<0.0001**
BUN (mmol/L)	5.27 (1.52–10.32)	6.12 (2.41–11.82)	**<0.0001**
CRE (mmol/L)	65.49 (39.18–88.54)	70.53 (42.20–115.11)	**0.001**
Underlying diseases	14 (19.18%)	107 (68.15%)	**<0.0001**
Type 2 diabetes	3 (4.11%)	25 (15.92%)	**0.011**
Hypertension	3 (4.11%)	32 (20.38%)	**0.001**
Coronary heart disease	2 (2.74%)	9 (5.73%)	0.509
Cor pulmonale	0	2 (1.27%)	1.000
Emphysema	0	6 (3.82%)	0.180
Chronic bronchitis	0	18 (11.46%)	**0.003**
Fatty liver disease	5 (6.85%)	15 (9.55%)	0.498
Hypothyroidism	1 (1.37%)	0	0.317

Data are median (range) or *n* (%). Bold *p* values represent significant differences

^a^Laboratory test indices are presented in abbreviations: *ALT* alanine aminotransferase, *AST* aspartate aminotransferase, *TBIL* total bilirubin, *TC* total cholesterol, *TG* triglyceride, *GLU* blood glucose, *BUN* blood urea nitrogen, *CRE* creatinine

### Safety of the vaccination protocol

**Fig. 2:**
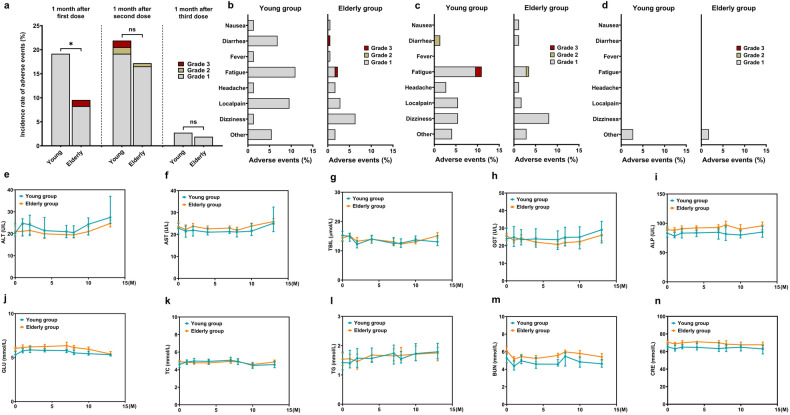
Safety evaluation. The incidence of adverse events within 1 month after each dose of COVID-19 vaccine (**a**) and the proportion of each adverse event to the overall in two groups within 1 month after the first dose (**b**), the second dose (**c**), and the booster (**d**). Changes in the biochemical indices in two groups at baseline and the 1st, 2nd, 4th, 7th, 8th, 10th, and 13th months (data of the 16th month was not detected due to the pandemic interference) shown in **e**–**n**. *P < 0.05; ns no significance; GGT γ-glutamyl transpeptidase; ALP alkaline phosphatase. Other adverse events included rash, cough, pharyngeal malaise, tinnitus, and insomnia

Within 1 month after priming of the inactivated vaccine (BBIBP-CorV), the incidence of adverse events in the elderly was significantly lower than that in the young (9.55% vs 19.18%, *P* = 0.041); after the second dose of BBIBP-CorV, the incidence of adverse events was similar in the two groups (17.18% vs 21.92%, *P* = 0.393) (Fig. 2a). In total, only four cases of grade 3 adverse events were reported; two cases in the elderly group after priming (one case of fatigue and one case of diarrhea) (Fig. 2b) and two cases in the young group after the second dose (both cases of fatigue) (Fig. 2c). After administering the booster dose (ZF2001), the incidence of adverse events decreased significantly to <3% in both groups (2.74% vs 1.91%, *P* = 0.375), and no grade 2 or 3 adverse events were reported (Fig. 2a, d). Local pain and fatigue were common in young participants, whereas dizziness was prevalent in elderly participants (Fig. 2b,c). All adverse events showed recovery in the short term (nausea, fever, and diarrhea resolved within 24 h; fatigue, dizziness, and local pain resolved in ~72 h). During the entire vaccination protocol and follow-up period, the parameters of liver function (Fig. 2e–i), blood glucose (Fig. 2j), blood lipid (Fig. 2k,l), and kidney function (Fig. 2m, n) of the participants in both groups were within the normal range and remained stable.

### Immunogenicity of COVID-19 vaccines is weaker in the elderly

**Fig. 3:**
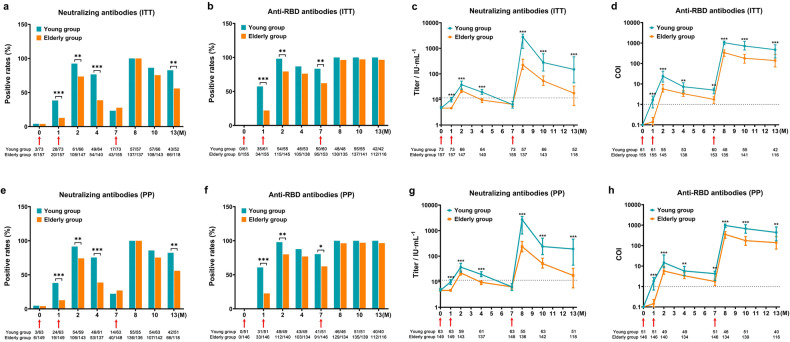
Positive rates and titers of neutralizing antibodies and anti-RBD antibodies. ITT analysis (**a**–**d**) and PP analysis (**e**–**h**) to illustrate antibody fluctuation trends. *P* values are shown only for significant differences, *P < 0.05, **P < 0.01, ***P < 0.001. Notes below each figure represented the number of participants tested at each sampling point and participants with positive test results. Red arrows indicated time points of vaccination. Dot lines represented the threshold of antibodies

In intention-to-treat (ITT) analysis, the positive rates of neutralizing antibodies in the elderly were lower than those in the young at the 1st month (12.74% vs 38.36%, *P* < 0.0001) and the peak of 2nd month (73.47% vs 92.42%, *P* = 0.002), while they also decreased rapidly at the 4th month (38.57% vs 76.56%, *P* < 0.0001) (Fig. 3a). After the booster, the positivity rate of neutralizing antibodies in the elderly group increased to 100%, comparable to that in the young group (Fig. 3a). However, the positive rate of neutralizing antibodies in the elderly population declined rapidly again, leading to a significant difference at the 13th month (55.93% vs 82.69%, *P* = 0.001) (Fig. 3a). The overall trend of anti-receptor binding domain (RBD) antibody positivity was similar to that of neutralizing antibodies, except that the positive rates in both groups remained high after administering the booster, with no significant differences between the two (Fig. 3b). Titers of neutralizing antibodies in the elderly were lower at the 1st month (4.6 IU/mL vs 9.96 IU/mL, *P* < 0.0001), the peak of the 2nd month (22.00 IU/mL vs 37.66 IU/mL, *P* < 0.0001), and the 4th month (9.49 IU/mL vs 19.34 IU/mL, *P* < 0.0001) than that in the young (Fig. 3c). A remarkable increase in neutralizing antibody titers was observed 1 month after administering the booster in both groups but it was still 12.38 times lower in the elderly than in the young (223.42 IU/mL vs 2764.86 IU/mL, *P* < 0.0001) (Fig. 3c). After 6 months of booster administration, the reduction in neutralizing antibodies in elderly and young people was basically the same, with a relative disparity of 8.51 times (17.61 IU/mL vs 149.87 IU/mL, *P* < 0.0001) (Fig. 3c). The titers of anti-RBD antibodies showed a similar trend (Fig. 3d). Per-protocol (PP) analysis showed results consistent with ITT analysis (Fig. 3e–h), indicating that the elderly had a relatively poor ability to maintain antibody levels, even if they received booster doses.

### Gender, age, and underlying diseases are negatively associated with antibody production in the elderly

Univariate and multivariate logistic regression analysis was performed to evaluate the factors affecting antibody production in elderly individuals (Table 2 and Supplementary Tables 2–7). Specifically, in the 2nd month, underlying diseases were independent risk factors for the production of neutralizing antibodies, with 77.2% reduction in the rate of neutralizing antibody production in elderly people (odds ratio [OR] 0.228, 95% confidence interval [CI] 0.094–0.550, *P* = 0.001) (Table 2). In the 7th month, age and underlying diseases were identified as risk factors for the production of neutralizing antibodies, with 11.2% and 62.3% reduction of the neutralizing antibody production rate, respectively (OR 0.888, 95% CI 0.815–0.967, *P* = 0.007 and OR 0.377, 95% CI 0.167–0.849, *P* = 0.019, respectively) (Table 2). And in the 2nd and 7th months, male gender and underlying diseases were identified as risk factors for the production of anti-RBD antibodies (Table 2). Our results suggesting that elderly individuals with male gender, advanced age, and underlying diseases exhibited lower levels of antibody production after vaccination.

**Table 2:** Factors influencing neutralizing and anti-RBD antibody production in the elderly

Time points	Variables	OR (95% CI)	*P* value
Neutralizing antibodies			
Month 2	Underlying diseases: combine (vs not combine)	0.228 (0.094–0.550)	0.001
Month 7	Age	0.888 (0.815–0.967)	0.007
	Underlying diseases: combine (vs not combine)	0.377 (0.167–0.849)	0.019
Anti-RBD antibodies			
Month 2	Gender: female (vs male)	2.576 (1.046–6.342)	0.040
	Underlying diseases: combine (vs not combine)	0.361 (0.144–0.903)	0.029
Month 7	Gender: female (vs male)	2.653 (1.257–5.600)	0.010
	Underlying diseases: combine (vs not combine)	0.398 (0.191–0.828)	0.014

Data were analyzed using multivariate logistic regression

### Antibody levels before infection are associated with symptoms severity after infection in the elderly

**Fig. 4:**
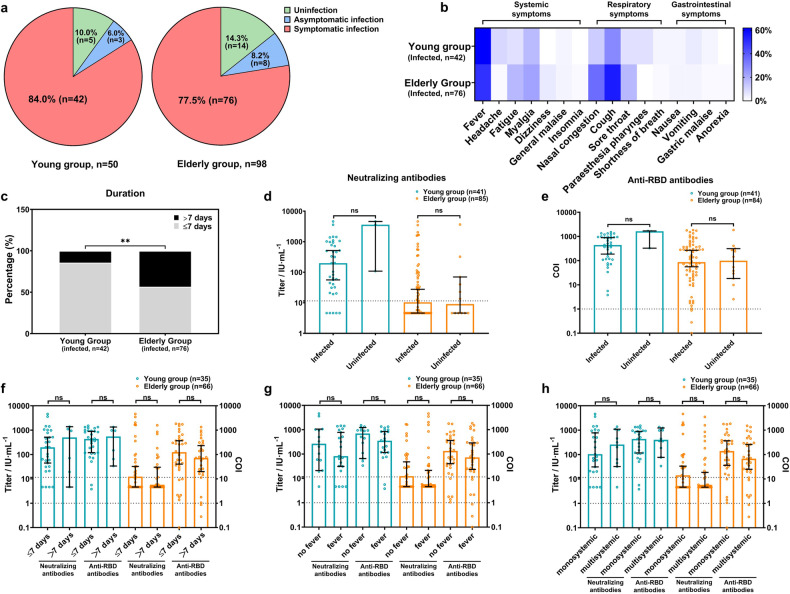
The relationship between breakthrough infection rates or symptoms and pre-infection antibody levels. The information on the breakthrough infection (**a**), the symptom types (**b**), and the symptom durations (**c**) were collected from 50 young and 98 elderly participants in the cohort. The association between pre-infection antibodies and infection status is presented in (**d**) and (**e**), whereas the relationship between pre-infection antibodies and symptoms is depicted by durations (**f**), accompanying fever (**g**), and symptom complexity (**h**) (monosystemic symptoms: only systemic, respiratory, or gastrointestinal symptoms; multisystemic symptoms: at least two of those three above). Data were analyzed using the chi-square test and Mann–Whitney *U* test. **P < 0.01. ns no significance. Dot lines represented the threshold of antibodies

During the COVID-19 pandemic, at the end of 2022, 50 young and 98 elderly participants were followed up. The baseline characteristics did not significantly differ between the lost and follow-up groups (data not shown). Forty-five young (90.0%) and 84 elderly (85.7%) participants were infected, showing no statistically significant differences (*P* = 0.461) (Fig. 4a). Among them, three young (6.0%) and eight elderly (8.2%) participants were asymptomatic, and no severe cases were reported (Fig. 4a). Young participants typically experienced fever, whereas elderly participants had both fever and cough (Fig. 4b). There were significantly more elderly participants with symptoms lasting longer than 1 week than young participants (43.42% vs 16.67%, *P* = 0.001) (Fig. 4c). There was no significant difference in the pre-infection antibody titers between the infected and uninfected participants in either group (Fig. 4d,e). Elderly individuals with lower antibody levels were having a potential risk of extended symptom duration, accompanied by fever, and showed multisystemic symptoms; even though the difference was not statistically significant (Fig. 4f–h). The demographic data of the two groups of participants are presented in Supplementary Table 8. Antibody levels before infection may play an important role in the severity of breakthrough infection symptoms in the elderly.

### Changes in antibodies before and after infection

**Fig. 5:**
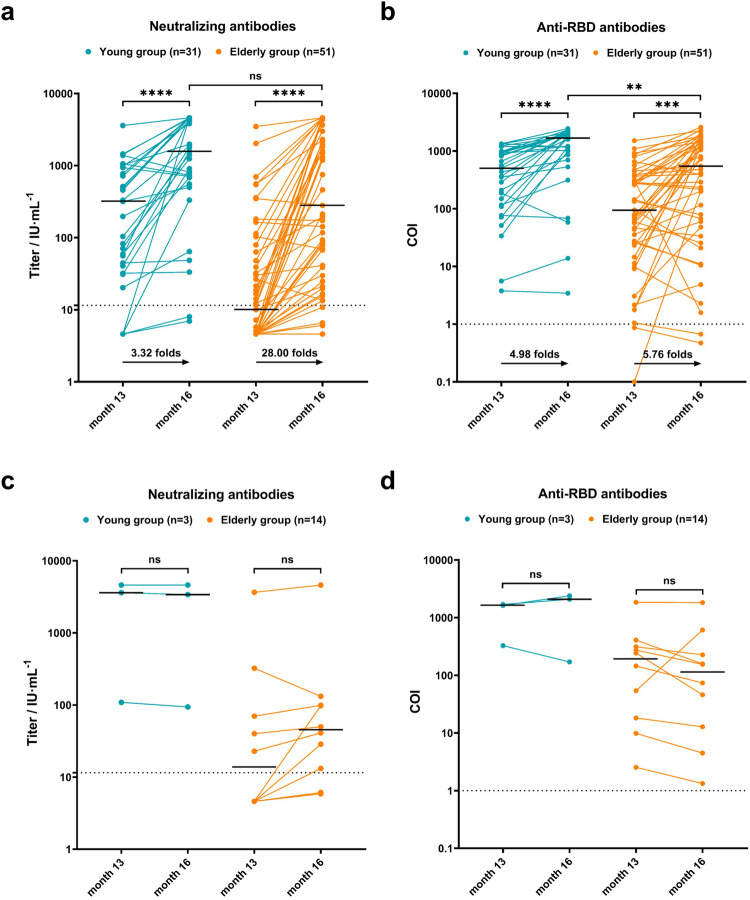
Analysis of changes in antibodies before and after infection. Changes in antibody levels were depicted in (**a**) and (**b**) for infected participants, and in (**c**) and (**d**) for uninfected participants. Data were analyzed using the Mann–Whitney *U* test. **P < 0.01, ***P < 0.001, ****P < 0.0001. ns no significance. Bold black horizontal lines represented the median values of antibody titers. Dot lines represented the threshold of antibodies

The levels of neutralizing antibodies were significantly higher in both young and elderly patients with breakthrough infections than those before infection (*P* < 0.0001) (Fig. 5a). Although the level of neutralizing antibodies in the elderly after infection was lower than that in the young, the difference was not statistically significant (Fig. 5a). Similarly, the post-infection anti-RBD antibody levels in elderly and young people with breakthrough infection were significantly higher (*P* < 0.0001 and *P* = 0.0002, respectively) (Fig. 5b). However, the anti-RBD antibody levels after breakthrough infection were still significantly lower in the elderly than in the young patients (*P* = 0.0012) (Fig. 5b). Interestingly, the level of neutralizing antibodies in the elderly after infection was 28.00-fold higher than that before infection, which was much higher than the 3.32-fold increase in young individuals. The increase in anti-RBD antibodies was similar in the elderly and young groups (4.98- and 5.76-fold, respectively). In the uninfected participants, there was no significant difference between the pre- and post-pandemic antibody levels (Fig. 5c, d).

### The production and protection of virus-specific neutralizing antibodies

**Fig. 6:**
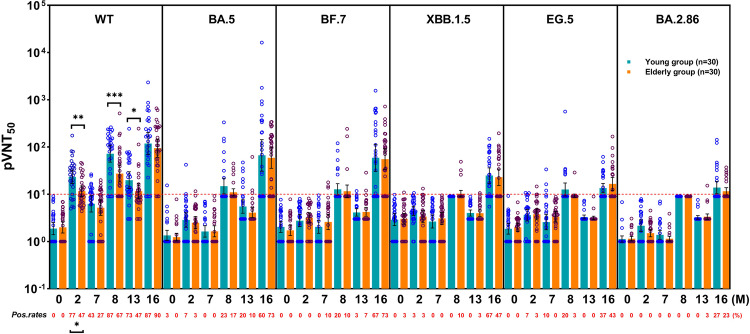
Positive rates and titers of different virus-specific neutralizing antibodies. Pseudovirus 50% neutralization titers (pVNT_50_) were detected in the two groups at baseline and 2, 7, 8, 13, and 16 months and are represented as geometric means in the column chart; the antibody-positive rates are indicated by the red number at the bottom of the figure. Only significant differences are marked with *P* values. The red dot line represents the antibody-positive threshold. Data were analyzed using the chi-square test and Mann–Whitney *U* test. *P < 0.05, **P < 0.01, ***P < 0.001. WT wild-type SARS-CoV-2 D614G; pos. rates positive rates

Thirty young and 30 elderly individuals were randomly selected from the breakthrough infection cohort for the pseudovirus neutralization assay. The results showed that 1 month after the 2nd dose, the positivity rate for wild-type SARS-CoV-2 D614G (WT)-specific neutralizing antibodies in the young group was significantly higher than that in the elderly group (77% vs 47%, *P* = 0.033). However, after receiving the booster dose and at the post-breakthrough infection, no significant differences were observed between the two groups. One month after the booster dose, the positivity rates for BA.5- and BF.7-specific neutralizing antibodies increased. After the breakthrough infection, all types of variant-specific neutralizing antibodies showed a certain positivity. However, compared with those of BA.5- and BF.7-, the positivity rate for XBB.1.5-specific neutralizing antibodies was low, and the positivity rates for EG.5- and BA.2.86-specific neutralizing antibodies were lower (Fig. 6).

The geometric mean of pseudovirus 50% neutralization titers (pVNT_50_) significantly differed only in the WT-specific neutralizing antibody assay at 1 month after the 2nd dose (25.48 vs 9.53, *P* = 0.003), 1 month after the booster (105.80 vs 22.14, *P* < 0.001), and pre-breakthrough infection (14.16 vs 9.20, *P* = 0.016). Notably, after the booster dose, BA.5- and BF.7-specific neutralizing antibody titers of some participants in both groups reached high levels; after the breakthrough infection, these titers were comparable to those of WT. For XBB.1.5, EG.5, and BA.2.86, very few positive neutralizing responses were observed during the vaccination process, and the titers did not reach the WT level, even after breakthrough infection (Fig. 6). In addition, analysis of symptomatic patients revealed that although most individuals had negative neutralizing responses to BA.5 and BF.7 before breakthrough infection, more cases of higher pVNT_50_ levels were observed in both groups of patients with short symptom duration and monosystemic symptoms and in the elderly population without fever (Supplementary Fig. 1).

Meanwhile, except at baseline, during the entire follow-up process, titers of WT-specific neutralizing antibodies in the entire cohort showed significantly strong positive correlations with pVNT_50_ levels (Supplementary Fig. 2). Correlations between the young group and entire cohort were similar; however, the elderly group showed a strong correlation only after the booster (Supplementary Fig. 2). Additionally, in the corresponding group, only the pVNT_50_s of BA.5- and BF.7-specific neutralizing antibodies showed weak but significant correlations with pVNT_50_s of the WT-specific neutralizing antibodies after the booster and before the pandemic (Supplementary Figs. 3 and 4). After the pandemic, all pVNT_50_s of variant-specific neutralizing antibodies, including XBB.1.5-, EG.5-, and BA.2.86-, showed strong significant positive correlations with the pVNT_50_s of WT-specific neutralizing antibodies (Supplementary Fig. 5).

## Discussion

The fragile immune system and the threat of SARS-CoV-2 variants make the elderly prone to a high risk of infection, hospitalization, and even death in the post-COVID era.^16,17^ Studies have shown that vaccines can effectively prevent severe COVID-19 cases due to variant infection.^18^ The WHO recommended a third booster dose for people who received the initial two doses 5 months ago or the adenovirus vaccine 2 months ago,^19–21^ for maintaining protective effects. Unfortunately, the coverage of primary series and booster vaccination for individuals aged 70 years and even older remains insufficient in China.^14^

In the initial phase of the COVID-19 pandemic, several clinical trials were undertaken to assess different types of vaccines against SARS-CoV-2, including whole-virus inactivated, adenovirus vector-based, recombinant protein-based, mRNA, and DNA vaccines, etc.^22–26^ As the pandemic progressed, attempts were made to combine homologous or heterologous vaccinations to improve the immunogenicity and efficacy of the vaccination protocol, while maintaining safety.^27–29^ The efficacy of these vaccines against SARS-CoV-2 variants and their ability to protect vulnerable populations have also received significant attention.^30–32^ A follow-up study conducted by Parry et al. for 8 months confirmed that both mRNA (BNT162b2) and adenovirus vaccines (ChAdOx1) not only have strong immunogenicity in elderly individuals but also can induce differential humoral and cellular immunity.^33^ Despite several other vaccine evaluation trials also focusing on the elderly population;^34–36^ currently, no prospective, real-world study data on the long-term effects of COVID-19 vaccination in this demographic are available.

Our study showed that two doses of BBIBP-CorV followed by booster ZF2001 were well tolerated, and the incidence of adverse events was low in the elderly. Very few cases of grade 3 adverse events have been reported. All adverse events resulted in rapid recovery without medical intervention. Moreover, multiple laboratory test indices remained stable during a follow-up period of up to 13 months. Our findings are consistent with those of several studies suggesting that the elderly may experience fewer adverse events following COVID-19 vaccination than younger people.^37,38^ Vaccine-related systemic adverse events are related to immune responses, and the incidence of early adverse events is low if the immune response is weak.^39^ Lower antibody levels in the elderly after vaccination also confirm this finding.

In our immunogenicity investigation, by confirming the consistency between ITT and PP analyses, two results were noteworthy. The first result we obtained was that at all follow-up time points, the antibody levels were significantly lower and decreased rapidly in the elderly. Based on WHO’s “5-month interval” principle for booster injection, a shorter interval of 3–4 months may help the elderly maintain adequate humoral immune response to alleviate symptoms of the COVID-19 breakthrough infection. Second, our results showed that the neutralizing antibody titers of elderly participants in the 1st month after the booster were 10 times higher than those in the 1st month after the second dose, whereas, in another study of a 3-dose COVID-19 inactivated vaccine protocol, there was only a 3.69-fold increase after the third dose.^40^ It is suggested that the sequential heterologous recombinant COVID-19 vaccine booster was more effective in reactivating the weakened immunological memory than that of the sequential homologous vaccine in the elderly. In addition, since the ZF2001 booster led to fewer adverse events than BBIBP-CorV, the recombinant protein vaccine may be a viable candidate for the second dose. A weak anti-RBD antibody production ability for male participants, which is compatible with the previous reports,^41^ was observed in our elderly cohort, and gender-specific behaviors, genetic and hormonal factors, and sex differences in biological pathways related to SARS-CoV-2 infection may lead to this result.^42^ At present, the severe infection of COVID-19 in the elderly is mainly due to the gradual aging of the immune system with increasing age, that is, immunosenescence, which can be caused by the weakened response to inflammatory stimuli, reduced migration and phagocytic ability of dendritic cells, the low reactivity of late differentiated immune cells, and atrophy of lymph nodes.^43^ Increased incidence of combined underlying diseases, especially type 2 diabetes,^44^ also negatively impacts the immune system in the elderly. An appropriate immunopotentiator dosage is recommended to address the adverse effects of these risk factors.

In our breakthrough infection cohort, there were no severe cases or deaths in the elderly population. In addition to the reduced virulence due to viral mutations, the protective efficacy of our vaccination strategy may also play an important role. Nevertheless, symptom duration in the elderly was significantly longer, and the range of respiratory system symptoms was wider. This may be related to the lower antibody levels before breakthrough infection in the elderly than in the young population. Further analysis demonstrated that elderly individuals with lower antibody titers were more prone to have longer symptom duration, greater likelihood of fever, and higher complexity of symptoms. This trend was not obvious in young people, which may be due to the high titers of neutralizing and anti-RBD antibodies before the breakthrough infection. However, a larger cohort will be required for confirmation.

When using vaccination strategies to prevent COVID-19 breakthrough infection, it is important to ensure the maintenance of its SARS-CoV-2-specific memory repertoire, so that the body can mobilize rapid and strong anamnestic response to fight against the virus when it invades again.^45^ Studies that have started to concentrate on the elderly population’s hybrid immunity with series booster vaccinations have so far reported encouraging outcomes.^46^ To gather further testing and analysis evidence, more research is still required. Based on some rare evidence, elderly people may have long-term immunological memory of SARS-CoV-2,^47^ and according to the most recent research, the elderly who survived the COVID-19 outbreak have memory B cells that can facilitate the maintenance of anti-RBD antibodies.^48^

Neutralizing antibody levels in elderly individuals recover quickly and strongly after re-exposure to antigen epitopes, suggesting that immunological memory in the elderly can be strengthened. As there is a difference in immunological memory between booster and breakthrough infection, more evidence is needed. At the end of 2022, Omicron lineages BA.5 and BF.7 dominated the pandemic in China. The pseudovirus neutralization assay showed that different types of variant-specific neutralizing antibodies, especially BA.5 and BF.7, can be induced by two doses of BBIBP-CorV followed by the booster ZF2001. For WT-specific neutralizing antibodies, higher production was highly correlated with stronger neutralizing ability, indicating that our vaccination protocol can simultaneously improve the quantity and quality of antibody production. In the elderly group, compared with inactivated vaccines, the booster dose greatly enhances the neutralizing ability of virus-specific antibodies rather than simply increasing antibody production. Although boosters may ultimately only activate existing immunological memory cells rather than create new memory cells, which are necessary to resist new variants,^49^ our data confirmed that boosters are indispensable for training the immune system in the elderly. After exposure to WT vaccines, the immune system of elderly people may also produce effective immune responses to BA.5 and BF.7 variants. Similarly, after experiencing breakthrough infection, some elderly patients can develop effective and specific responses to XBB.1.5, EG.5, and BA.2.86 that they have not been exposed to. Overall, the protective efficacy of vaccines can be expanded, and the closer the kinship of the variant, the greater its protective effect. These neutralizing antibodies may serve as crucial factors in the prevention of severe illnesses or even fatalities during future outbreaks. Vaccination with the WT SARS-CoV-2 may not induce immunological protection against SARS-CoV-2 variants in a large proportion of the population, especially the elderly. Receiving vaccines designed on the basis of variants may provide better protection; however, sequential boosters at shorter time intervals are still necessary. In view of a recent report, the overall incidence of COVID-19 reinfection in China has already reached 28.3%, and the longer the time from the first infection, the higher the incidence of reinfection,^50^ suggesting that the elderly still need multiple booster vaccinations after the global COVID-19 epidemic, even if some of them have a full vaccination history.

Our study had some limitations. First, the weak physical status of the elderly, the interference of the COVID-19 pandemic, and the long-term follow-up itself objectively led to the loss of follow-up and dropouts, which gradually decreased the cohort size. Second, owing to the sample consumption caused by clinical testing, our project did not include an in-depth, comprehensive analysis of virus-specific T-cell immunity, which weakens the impact to some extent. Lastly, without an inactivated vaccine booster control cohort, we could not directly demonstrate the advantages of a heterologous recombinant COVID-19 vaccine booster.

In conclusion, with a long-term follow-up study in this prospective observational clinical cohort, we confirmed that the heterologous vaccination protocol not only enhanced immunogenicity while ensuring safety but also elicited promising protective efficacy. Due to the weakened immune response in elderly individuals, it is pivotal for them to receive prompt and appropriate SARS-CoV-2 variant vaccines, depending on the prevalence of the variant types. More importantly, according to our findings, administering multiple COVID-19 booster shots at short intervals in the elderly population may be a protective strategy. Further studies on better vaccine combinations and immune mechanisms are required.

## Materials and methods

### Study design

This was a prospective observational clinical cohort study conducted from July 29, 2021, to June 30, 2023, in Hunyuan County, Shanxi Province. This study was conducted according to the principles of the Declaration of Helsinki. The study protocol was approved by the Ethics Committee of the Fifth Medical Center of the PLA General Hospital and clinical registration was completed (NCT05012800). Signed informed consent was obtained from each participant prior to screening.

As shown in Fig. 1, all participants received two doses of inactivated vaccine at 21–28 days intervals (annotated as the 1-month interval for narrative convenience) and then received the third recombinant protein vaccine 6 months after the second dose. The first two doses were China’s Sinopharm COVID-19 inactivated vaccine BBIBP-CorV (Vero cells) containing 4 μg/0.5 mL in a vial. The third booster dose was Zhifei Longcom recombinant COVID-19 vaccine ZF2001 (CHO cells) containing 25 μg/0.5 mL in a vial. Both vaccines were adjuvanted by aluminum hydroxide. All the participants received the vaccine intramuscularly through a deltoid.

Follow-up visits were conducted at baseline and 1st, 2nd, 4th, 7th, 8th, 10th, 13th, and 16th months. Participant diary cards were established to record short-term adverse events within 1 month of receiving each dose of the vaccine, and long-term adverse events at the 4th, 7th, 10th, and 13th months. Blood samples were collected for laboratory testing and antibody titer determination. The COVID-19 outbreak and breakthrough infection occurred between the 13th and 16th months of follow-up. SARS-CoV-2 infection was diagnosed by nucleic acid or antigen testing.

The main endpoints of this study were safety profiles within 1 month after each vaccination dose, including the incidence of adverse events, liver and kidney function, blood glucose and lipid levels, and routine blood indices. The definition and grade of adverse events were evaluated according to the Common Terminology Criteria for Adverse Events, version 5.0. Briefly, events were rated as grade 1 (mild), asymptomatic or mild symptoms, clinical or diagnostic observations only, intervention not indicated; grade 2 (moderate), minimal, local, or non-invasive intervention indicated, limiting age-appropriate instrumental activities of daily living (ADL); and grade 3 (severe or medically significant but not immediately life-threatening), indication for hospitalization or prolongation of hospitalization, disabling and limiting self-care ADL. The secondary endpoints were immune protection of the participants, specifically the titers of neutralizing and anti-RBD antibodies at 1, 3, and 6 months after each vaccination dose.

### Enrollment criteria for participants

Eligible participants were those provided (1) signed informed consent; (2) in females, the urine pregnancy test was negative; (3) at least 6 months of follow-up was completed; (4) HBsAg, anti-HCV, HIV, TPHA screening was negative; (5) armpit temperature was ≤37.0 °C; (6) the young group was aged 18–59 years, and the elderly group between 60 and 80 years.

Exclusion criteria were as follows: (1) in females, the urine pregnancy test positive; (2) pregnant or lactating women; (3) known allergies to certain components of these two vaccines; (4) patients with serious chronic diseases or advanced diseases such as high blood pressure, diabetes, asthma, thyroid disease, etc. which cannot be controlled by drugs; (5) those suffering from thrombocytopenia, hemorrhagic disease or thrombotic disease; (6) those with congenital or acquired angioedema/neuroedema; (7) those with a history or family history of convulsions, epilepsy, encephalopathy, other progressive neurological diseases, and psychiatric disorders; (8) lymphadenopathy, (9) lymphoma, leukemia, and other systemic malignancies; (10) autoimmune disease, and (11) chronic diseases with cute exacerbation or acute infectious diseases and fever.

### Antibody detection

The chemiluminescent microparticle immunoassay was used to detect SARS-CoV-2 spike protein-neutralizing antibodies titers (detection range: 4.6–4600 IU/mL, positive threshold: 11.5 IU/mL, performed by Wan Tai Kairui Biotechnology Co., Ltd. with the protocol of the 2019-nCoV Neutralizing Antibody Detection Kit) and anti-RBD antibodies titers (positive threshold: 1.0 cut-off index, performed by Wan Tai Kairui Biotechnology Co., Ltd. with the protocol of the 2019-nCoV Antibody Detection Kit) in human plasma samples. The sample, 2019-nCoV recombinant antigen-coated magnetic particles, and reaction diluent were mixed. After washing, acridine ester labeled with 2019-nCoV recombinant antigen (or anti-human lgG antibody) was added to the reaction system to form a complex. Pre-excitation and excitation solutions were added after washing the samples again. Chemiluminescence reaction signals were measured and expressed in relative luminescent units (RLUs), and antibody titers in the samples were proportional to the RLU.

According to the protocols of JOINN Beijing Technology Testing Co., Ltd., pseudovirus neutralizing assay was used to detect the neutralizing activity of WT-, BA.5-, BF.7-, XBB.1.5-, EG.5- and BA.2.86-specific neutralizing antibodies against the SARS-CoV-2 spike protein. The HIV lentivirus vector containing the surface-expressing SARS-CoV-2 spike protein was incubated with HEK293T-ACE2 cells in DMEM. Neutralization of the pseudovirus was measured after serial dilution of the sample, and the results were expressed as reciprocal titer of the sample required to reduce the RLUs by 50% compared with the control group. The positive threshold was set to 10.

### Statistical analysis

All statistical analyses were performed using IBM SPSS (version 26). Participants in both groups who had received all three doses of the COVID-19 vaccine were included in the ITT analysis, and those who completed more than six follow-up visits were included in the PP analysis. The Kolmogorov–Smirnov test was used to evaluate the distribution types. Non-normally distributed and ordered data are represented by the median (range). Measurement of pVNT_50_s is represented by geometric means. Pearson’s chi-square test, continuity correction, and Fisher’s exact test were used to check the proportion of the count data. The Mann–Whitney *U* test and paired *t*-test were used to compare differences between groups, the Wilcoxon rank-sum test was used to compare intragroup differences, and the nest *t*-test was used to compare unmatched groups. We displayed the median values of each group in the figures using GraphPad Prism 8 and estimated the differences based on the 95% CI. Baseline characteristics were screened using univariate logistic regression, and the significantly different factors (*P*_value < 0.1) were further analyzed using multivariate logistic regression. The analysis results are presented using an OR combined with a 95% CI (lower–upper) in the table. In correlation analysis, the Pearson correlation test was used to process normally distributed datasets, while the Spearman rank correlation test was used to process non-normally distributed datasets. An absolute *R* value > 0.5 indicated strong correlation, whereas an absolute *R* value ≤ 0.5 indicated weak correlation. The hypothesis test was bilateral, and a *P*_value < 0.05 was considered statistically significant.

### Supplementary information

Supplementary Materials

Data S1 - protocol

## Data Availability

All of the data generated and analyzed during this study are included in our manuscript.
