# Supplementary material for: Safety, immunogenicity and protective effect of sequential vaccination with inactivated and recombinant protein COVID-19 vaccine in the elderly: a prospective longitudinal study
Source: Signal Transduct Target Ther. 2024 May 13;9:129. doi: 10.1038/s41392-024-01846-9 (PMC11091094; doi:10.1038/s41392-024-01846-9)
Supplement: Supplementary file 1 — Supplementary Materials [file 41392_2024_1846_MOESM1_ESM.docx]

Supplementary Materials for

**Safety, immunogenicity and protective effect of sequential vaccination with inactivated and recombinant protein COVID-19 vaccine in the elderly: a prospective longitudinal study**

**AUTHORS**

Hong-Hong Liu, Yunbo Xie, Bao-Peng Yang, Huan-Yue Wen, Peng-Hui Yang, Jin-E Lu, Yan Liu, Xi Chen, Meng-Meng Qu, Yang Zhang, Wei-Guo Hong, Yong-Gang Li, Junliang Fu and Fu-Sheng Wang

Correspondence to: [fjunliang@163.com](mailto:fjunliang@163.com) and [fswang302@163.com](mailto:fswang302@163.com).

**This PDF file includes:**

Figures S1 to S5

Tables S1 to S8


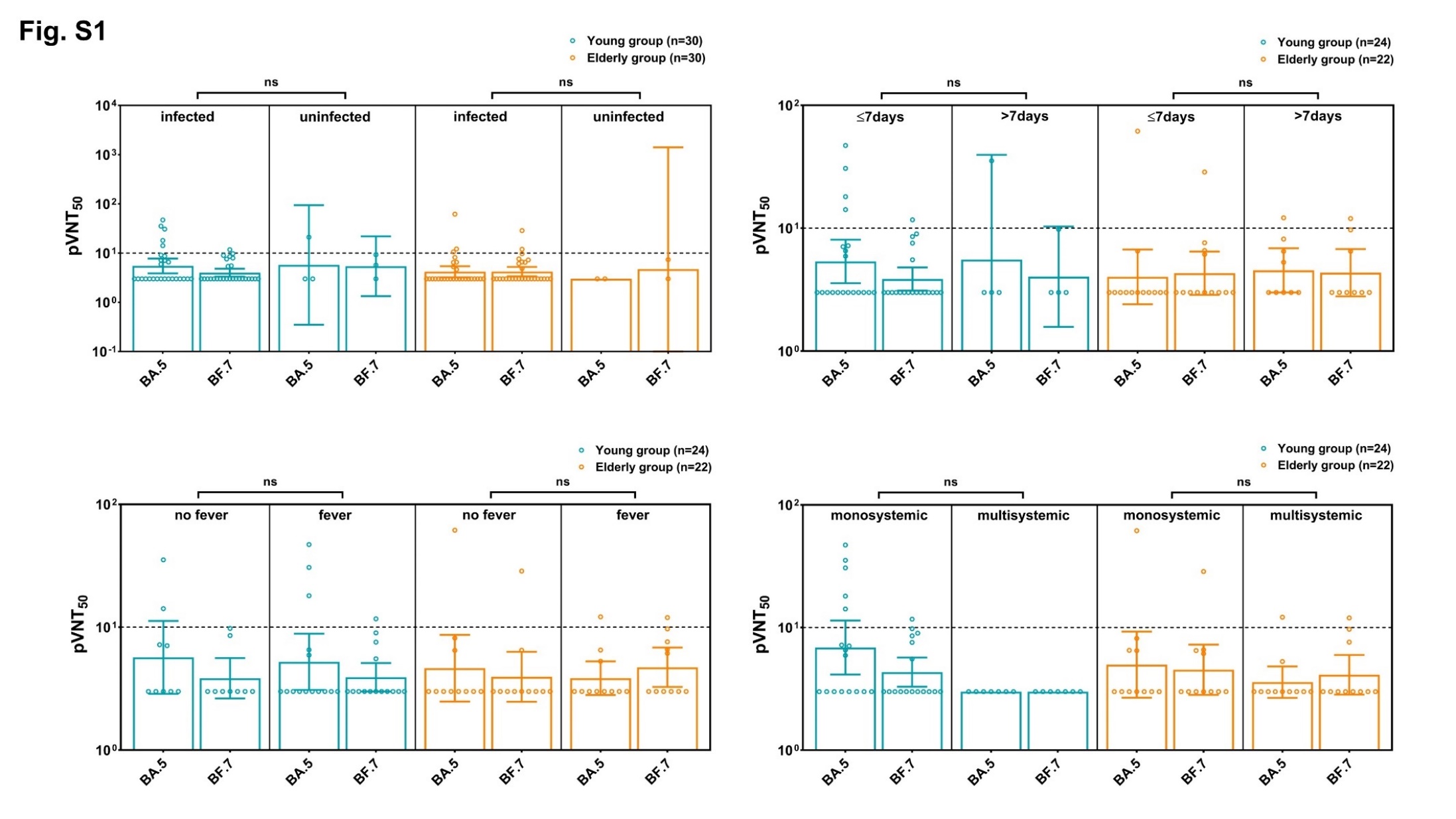


Figure. S1. The relationship between breakthrough infection status or symptoms and pre-infection virus-specific neutralizing antibodies. Pseudovirus 50% neutralization titers (pVNT_50_s) were analyzed in the two groups at the pre-infection timepoint (the 13^th^ month) and are represented as geometric means. The association between pre-infection virus-specific neutralizing antibodies and infection status, or symptoms which are depicted by durations, accompanying fever, and symptom complexity, are presented. Data were analyzed using the nest t-test. Dot lines represented the threshold of antibodies. ns: no significance.


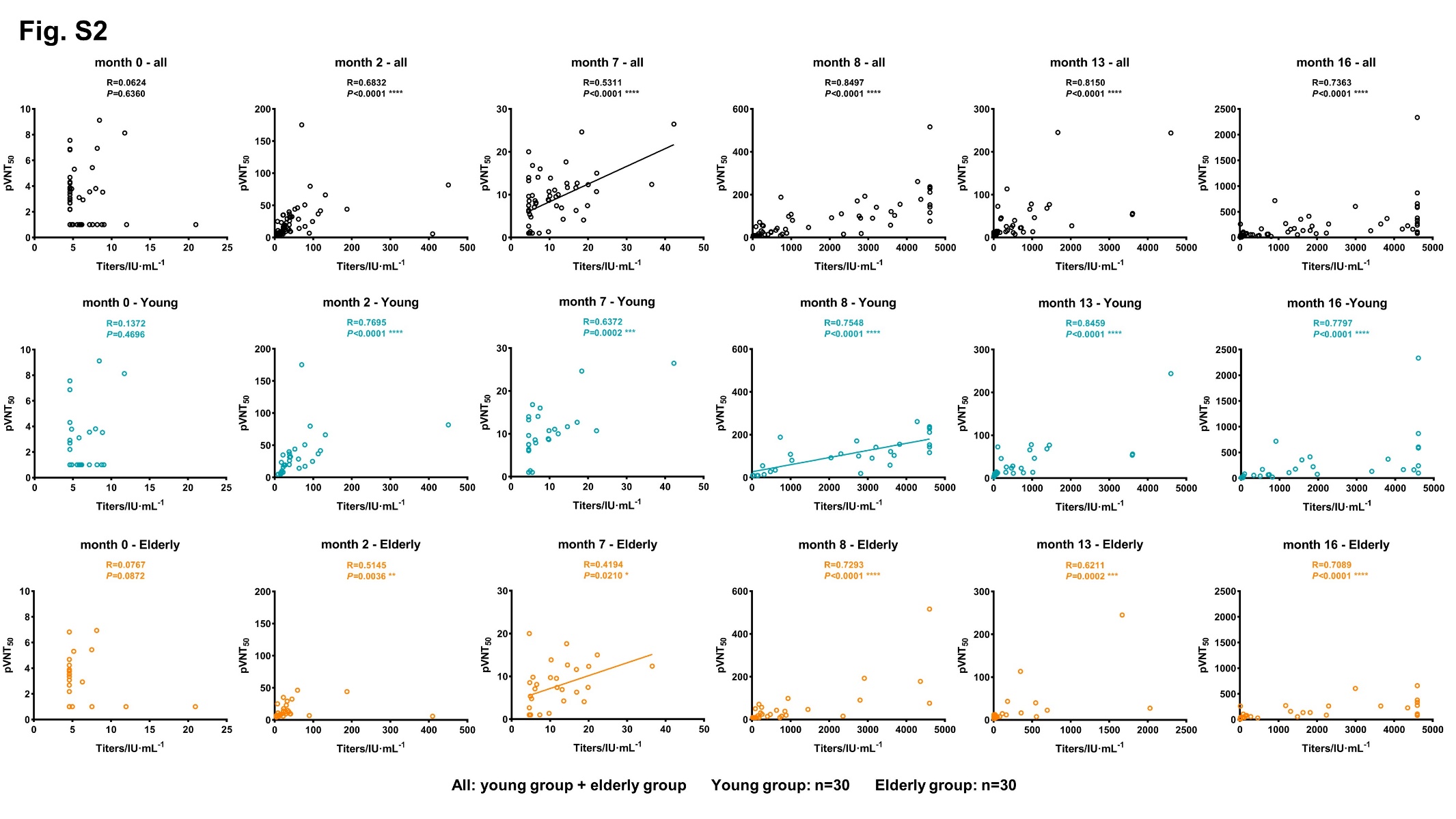


Figure. S2. Correlations analysis of the wild-type SARS-CoV-2 D614G (WT)-specific neutralizing antibodies between concentrations and neutralizing abilities. The correlation between concentrations (titers) and neutralizing capacities (pVNT_50_s) was analyzed at the baseline and the 2^nd^, 7^th^, 8^th^, 13^th^, and 16^th^ months of follow-up. The hollow dots represent each individual. A trend line indicates normally distributed data. Data were analyzed using the Pearson correlation test or the Spearman rank correlation test. *: *P* < 0.05, **: *P* < 0.005, ***: *P* < 0.001, ****: *P* < 0.0001.


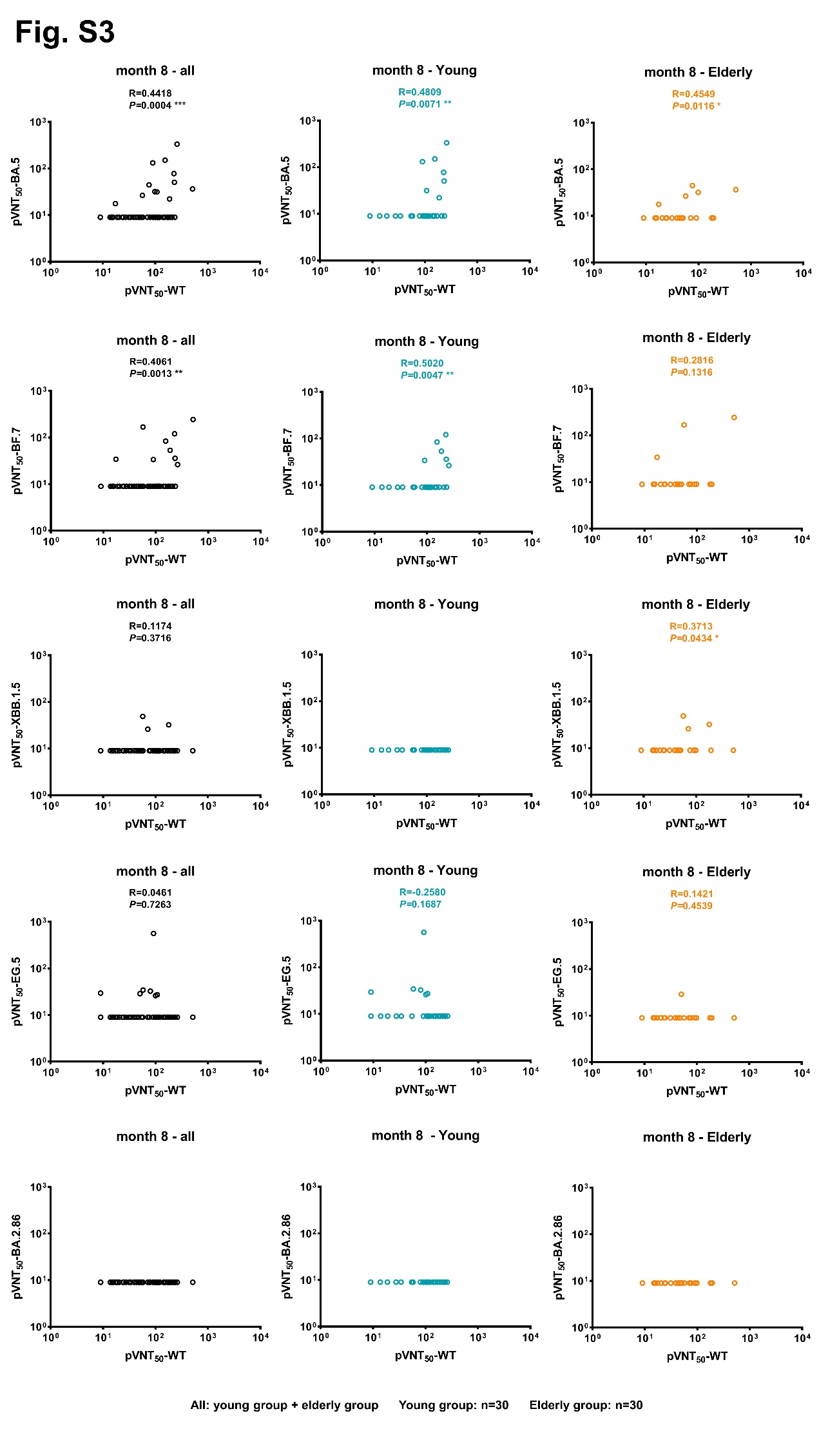


**Figure. S3. Correlations analysis of neutralization abilities between the WT-specific and variant-specific neutralizing antibodies after the booster.** The pVNT_50_s of the wild-type (WT)-specific and variant-specific neutralizing antibodies at the 8^th^ month are presented, and the corresponding correlations between them were analyzed. The hollow dots represent each individual. Data were analyzed using the Spearman rank correlation test. *: *P* < 0.05, **: *P* < 0.005, ***: *P* < 0.001.


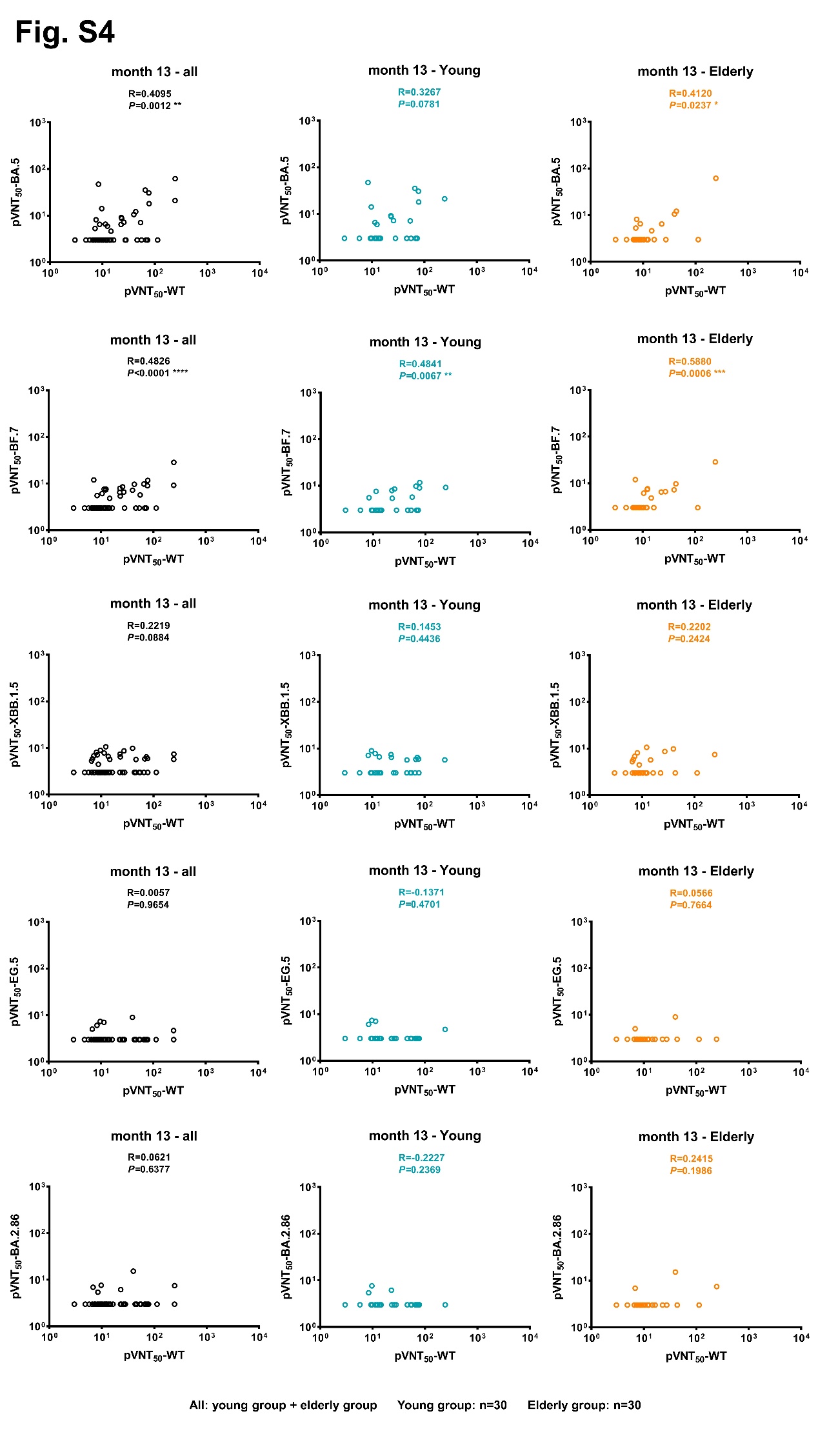


Figure. S4. Correlations analysis of neutralization abilities between the WT-specific and variant-specific neutralizing antibodies before the pandemic. The pVNT_50_s of the WT-specific and variant-specific neutralizing antibodies at the 13^th^ month are presented, and the corresponding correlations between them were analyzed. The hollow dots represent each individual. Data were analyzed using the Spearman rank correlation test. *: *P* < 0.05, **: *P* < 0.005, ***: *P* < 0.001, ****: *P* < 0.0001.


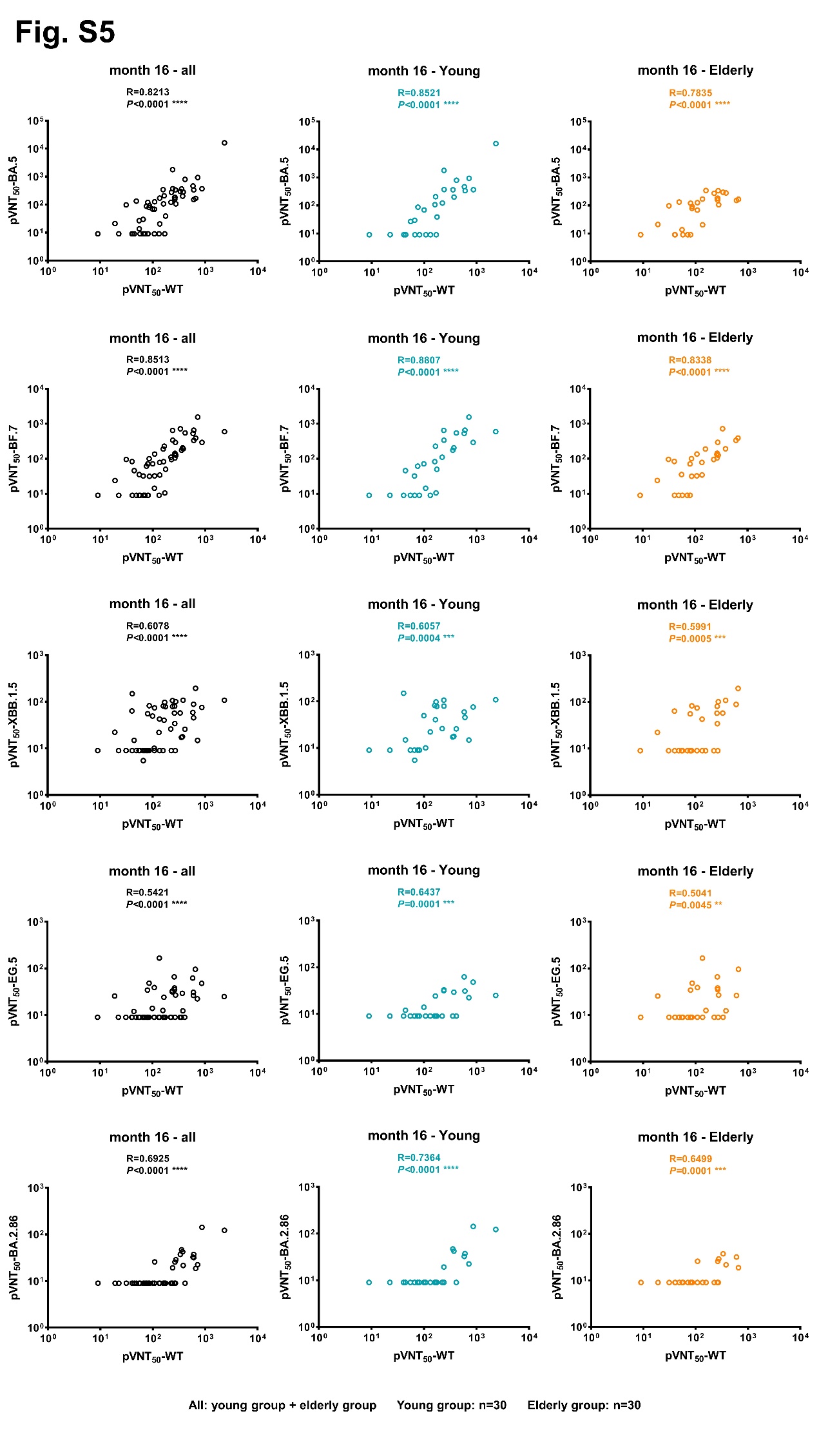


Figure. S5. Correlations analysis of neutralization abilities between the WT-specific and variant-specific neutralizing antibodies after the pandemic. The pVNT_50_s of the WT-specific and variant-specific neutralizing antibodies at the 16^th^ month are presented, and the corresponding correlations between them were analyzed. The hollow dots represent each individual. Data were analyzed using the Spearman rank correlation test. **: *P* < 0.005, ***: *P* < 0.001, ****: *P* < 0.0001.

**Table S1. The reasons and corresponding numbers of loss to follow-up for the participants in this study.**

| ***Follow-up visit*** | | | ***Baseline*** | ***Month 1*** | ***Month 2*** | ***Month 4*** | ***Month 7*** | ***Month 8*** | ***Month 10*** | ***Month 13*** | ***Month 16*** |
| --- | --- | --- | --- | --- | --- | --- | --- | --- | --- | --- | --- |
| **Elderly** | **Sample size** | | **157** | **157** | **147** | **140** | **155** | **137** | **143** | **118** | **98** |
|  | Reasons for loss to follow-up | COVID-19 epidemic lockdown^a^ | 0 | 0 | 2 | 2 | 0 | 5 | 3 | 6 | 0 |
|  |  | Out of the visit city^b^ | 0 | 0 | 3 | 5 | 0 | 5 | 4 | 11 | 12 |
|  |  | Personal illness^c^ | 0 | 0 | 2 | 3 | 0 | 4 | 2 | 3 | 0 |
|  |  | Lost contact^d^ | 0 | 0 | 3 | 4 | 2 | 5 | 4 | 12 | 21 |
|  |  | Withdrawal^e^ | 0 | 0 | 0 | 0 | 0 | 0 | 0 | 0 | 22 |
|  |  | Other reasons^f^ | 0 | 0 | 0 | 3 | 0 | 1 | 1 | 7 | 4 |
|  |  | Total | 0 | 0 | 10 | 17 | 2 | 20 | 14 | 39 | 59 |
| **Young** | **Sample size** | | **73** | **73** | **66** | **64** | **73** | **57** | **66** | **52** | **50** |
|  | Reasons for loss to follow-up | COVID-19 epidemic lockdown^a^ | 0 | 0 | 1 | 2 | 0 | 4 | 1 | 5 | 0 |
|  |  | Out of the visit city^b^ | 0 | 0 | 3 | 4 | 0 | 5 | 4 | 9 | 8 |
|  |  | Personal illness^c^ | 0 | 0 | 1 | 0 | 0 | 1 | 1 | 1 | 0 |
|  |  | Lost contact^d^ | 0 | 0 | 2 | 2 | 0 | 4 | 1 | 4 | 5 |
|  |  | Withdrawal^e^ | 0 | 0 | 0 | 0 | 0 | 0 | 0 | 0 | 7 |
|  |  | Other reasons^f^ | 0 | 0 | 0 | 1 | 0 | 2 | 0 | 2 | 3 |
|  |  | Total | 0 | 0 | 7 | 9 | 0 | 16 | 7 | 21 | 23 |

^a^Participants were unable to come for a follow-up visit as their residential area was temporarily placed on lockdown in 2022. ^b^Participants could not attend the follow-up visit because they lived in another city due to personal reasons. ^c^Participants failed to attend the follow-up visit due to their non-vaccination-associated illness, such as acute gallstones, lumbar disc herniation, fractures, and ankle sprains. ^d^Phone calls of participants are unanswered, rejected, or turned off more than 5 times in total during the scheduled follow-up visit. ^e^At this follow-up point, SARS-CoV-2 infection was classified as a category B infectious disease in China, and some participants were unwilling to continue cooperating with the follow-up. ^f^Participants were unable to attend the follow-up visit on schedule due to other reasons, such as taking care of their sick family members or refusing to take blood samples.

**Table S2. Univariate and multivariate logistic regression analysis of influencing factors for neutralizing antibody production in the elderly at the 2^nd^ month.**

|  | **Neutralizing antibodies - month 2 (n=134)** | | | |
| --- | --- | --- | --- | --- |
|  | **Univariate logistic regression** | | **Multivariate logistic regression** | |
| **Variables** | **OR (95% CI)** | ***P*_value** | **OR (95% CI)** | ***P*_value** |
| *Gender*: *female (v. male)* | 1.080 (0.500-2.336) | 0.844 |  |  |
| *Age* | 0.939 (0.868-1.015) | 0.113 |  |  |
| *BMI* | 1.006 (0.901-1.124) | 0.913 |  |  |
| *Underlying disease:* *combine (v. not combine)* | 0.228 (0.094-0.550) | 0.001 | 0.228 (0.094-0.550) | 0.001 |
| *ALT* | 1.012 (0.972-1.054) | 0.550 |  |  |
| *AST* | 1.004 (0.956-1.055) | 0.859 |  |  |
| *TBIL* | 1.021 (0.940-1.110) | 0.615 |  |  |
| *TC* | 1.318 (0.867-2.004) | 0.196 |  |  |
| *TG* | 1.087 (0.755-1.566) | 0.652 |  |  |
| *GLU* | 0.946 (0.824-1.085) | 0.427 |  |  |
| *BUN* | 1.047 (0.826-1.327) | 0.704 |  |  |
| *CRE* | 0.996 (0.968-1.024) | 0.762 |  |  |

For univariate and multivariate logistic regression, *P*_value less than 0.1 and 0.05 is considered significant, respectively. The significant OR (95% CI) and the corresponding *P*_value are highlighted in red.

**Table S3. Univariate and multivariate logistic regression analysis of influencing factors for anti-RBD antibody production in the elderly at the 2^nd^ month.**

|  | **Anti-RBD antibodies - month 2 (n=134)** | | | |
| --- | --- | --- | --- | --- |
|  | **Univariate logistic regression** | | **Multivariate logistic regression** | |
| **Variables** | **OR (95% CI)** | ***P*_value** | **OR (95% CI)** | ***P*_value** |
| *Gender*: *female (v. male)* | 2.094 (0.884-4.959) | 0.093 | 2.576 (1.046-6.342) | 0.040 |
| *Age* | 0.984 (0.907-1.069) | 0.709 |  |  |
| *BMI* | 1.092 (0.962-1.239) | 0.172 |  |  |
| *Underlying disease:* *combine (v. not combine)* | 0.439 (0.182-1.059) | 0.067 | 0.361 (0.144-0.903) | 0.029 |
| *ALT* | 1.008 (0.966-1.053) | 0.707 |  |  |
| *AST* | 1.002 (0.950-1.056) | 0.951 |  |  |
| *TBIL* | 1.044 (0.949-1.149) | 0.375 |  |  |
| *TC* | 0.974 (0.639-1.487) | 0.904 |  |  |
| *TG* | 0.914 (0.643-1.301) | 0.618 |  |  |
| *GLU* | 0.914 (0.794-1.054) | 0.216 |  |  |
| *BUN* | 1.114 (0.855-1.453) | 0.424 |  |  |
| *CRE* | 1.004 (0.974-1.036) | 0.797 |  |  |

For univariate and multivariate logistic regression, *P*_value less than 0.1 and 0.05 is considered significant, respectively. The significant OR (95% CI) and the corresponding *P*_value are highlighted in red.

**Table S4. Univariate and multivariate logistic regression analysis of influencing factors for neutralizing antibody production in the elderly at the 7^th^ month.**

|  | **Neutralizing antibodies - month 7 (n=146)** | | | |
| --- | --- | --- | --- | --- |
|  | **Univariate logistic regression** | | **Multivariate logistic regression** | |
| **Variables** | **OR (95% CI)** | ***P*_value** | **OR (95% CI)** | ***P*_value** |
| *Gender*: *female (v. male)* | 1.680 (0.803-3.516) | 0.169 |  |  |
| *Age* | 0.880 (0.814-0.952) | 0.001 | 0.888 (0.815-0.967) | 0.007 |
| *BMI* | 1.027 (0.924-1.142) | 0.616 |  |  |
| *Underlying disease:* *combine (v. not combine)* | 0.460 (0.218-0.970) | 0.041 | 0.377 (0.167-0.849) | 0.019 |
| *ALT* | 0.997 (0.959-1.037) | 0.883 |  |  |
| *AST* | 0.963 (0.904-1.026) | 0.246 |  |  |
| *TBIL* | 0.929 (0.855-1.009) | 0.082 | 0.916 (0.836-1.004) | 0.061 |
| *TC* | 1.271 (0.927-1.744) | 0.137 |  |  |
| *TG* | 1.171 (0.905-1.513) | 0.230 |  |  |
| *GLU* | 0.959 (0.856-1.075) | 0.471 |  |  |
| *BUN* | 0.822 (0.629-1.076) | 0.154 |  |  |
| *CRE* | 0.968 (0.941-0.996) | 0.025 | 0.975 (0.945-1.007) | 0.124 |

For univariate and multivariate logistic regression, *P*_value less than 0.1 and 0.05 is considered significant, respectively. The significant OR (95% CI) and the corresponding *P*_value are highlighted in red.

**Table S5. Univariate and multivariate logistic regression analysis of influencing factors for anti-RBD antibody production in the elderly at the 7^th^ month.**

|  | **Anti-RBD antibodies - month 7 (n=146)** | | | |
| --- | --- | --- | --- | --- |
|  | **Univariate logistic regression** | | **Multivariate logistic regression** | |
| **Variables** | **OR (95% CI)** | ***P*_value** | **OR (95% CI)** | ***P*_value** |
| *Gender*: *female (v. male)* | 2.554 (1.280-5.095) | 0.008 | 2.653 (1.257-5.600) | 0.010 |
| *Age* | 0.962 (0.900-1.028) | 0.253 |  |  |
| *BMI* | 1.067 (0.965-1.179) | 0.207 |  |  |
| *Underlying disease:* *combine (v. not combine)* | 0.506 (0.256-1.003) | 0.051 | 0.398 (0.191-0.828) | 0.014 |
| *ALT* | 0.984 (0.949-1.019) | 0.360 |  |  |
| *AST* | 1.015 (0.960-1.073) | 0.602 |  |  |
| *TBIL* | 0.973 (0.912-1.037) | 0.400 |  |  |
| *TC* | 1.050 (0.783-1.407) | 0.745 |  |  |
| *TG* | 1.069 (0.825-1.386) | 0.612 |  |  |
| *GLU* | 0.954 (0.868-1.049) | 0.329 |  |  |
| *BUN* | 0.800 (0.630-1.016) | 0.068 | 0.838 (0.648-1.085) | 0.180 |
| *CRE* | 0.985 (0.962-1.009) | 0.218 |  |  |

For univariate and multivariate logistic regression, *P*_value less than 0.1 and 0.05 is considered significant, respectively. The significant OR (95% CI) and the corresponding *P*_value are highlighted in red.

**Table S6. Univariate and multivariate logistic regression analysis of influencing factors for neutralizing antibody production in the elderly at the 13^th^ month.**

|  | **Neutralizing antibodies - month 13 (n=114)** | | | |
| --- | --- | --- | --- | --- |
|  | **Univariate logistic regression** | | **Multivariate logistic regression** | |
| **Variables** | **OR (95% CI)** | ***P*_value** | **OR (95% CI)** | ***P*_value** |
| *Gender*: *female (v. male)* | 1.462 (0.693-3.085) | 0.318 |  |  |
| *Age* | 0.928 (0.859-1.002) | 0.055 | 0.928 (0.859-1.002) | 0.055 |
| *BMI* | 0.950 (0.853-1.059) | 0.359 |  |  |
| *Underlying disease:* *combine (v. not combine)* | 0.617 (0.292-1.301) | 0.205 |  |  |
| *ALT* | 1.001 (0.997-1.004) | 0.615 |  |  |
| *AST* | 0.998 (0.972-1.024) | 0.870 |  |  |
| *TBIL* | 0.973 (0.910-1.040) | 0.417 |  |  |
| *TC* | 1.029 (0.714-1.483) | 0.878 |  |  |
| *TG* | 0.873 (0.642-1.187) | 0.386 |  |  |
| *GLU* | 0.939 (0.834-1.058) | 0.301 |  |  |
| *BUN* | 1.139 (0.878-1.478) | 0.326 |  |  |
| *CRE* | 0.978 (0.951-1.006) | 0.130 |  |  |

For univariate and multivariate logistic regression, *P*_value less than 0.1 and 0.05 is considered significant, respectively. The significant OR (95% CI) and the corresponding *P*_value are highlighted in red.

**Table S7. Univariate and multivariate logistic regression analysis of influencing factors for anti-RBD antibody production in the elderly at the 13^th^ month.**

|  | **Anti-RBD antibodies - month 13 (n=114)** | | | |
| --- | --- | --- | --- | --- |
|  | **Univariate logistic regression** | | **Multivariate logistic regression** | |
| **Variables** | **OR (95% CI)** | ***P*_value** | **OR (95% CI)** | ***P*_value** |
| *Gender*: *female (v. male)* | 2.893 (0.292-28.677) | 0.364 |  |  |
| *Age* | 1.042 (0.852-1.275) | 0.688 |  |  |
| *BMI* | 0.999 (0.747-1.335) | 0.993 |  |  |
| *Underlying disease:* *combine (v. not combine)* | 0.000 (0.000-?)* | 0.997 |  |  |
| *ALT* | 1.001 (0.991-1.010) | 0.892 |  |  |
| *AST* | 1.041 (0.909-1.193) | 0.561 |  |  |
| *TBIL* | 1.114 (0.876-1.417) | 0.379 |  |  |
| *TC* | 0.784 (0.297-2.073) | 0.624 |  |  |
| *TG* | 0.825 (0.411-1.656) | 0.588 |  |  |
| *GLU* | 1.038 (0.716-1.505) | 0.843 |  |  |
| *BUN* | 0.873 (0.461-1.652) | 0.676 |  |  |
| *CRE* | 1.004 (0.932-1.082) | 0.909 |  |  |

*Unreasonable logistic regression model due to insufficient anti-RBD antibody-negative participants in the 13^th^ month. However, considering the completeness of the analysis and the validity of other results in the same studies, it is still necessary to display them.

For univariate and multivariate logistic regression, *P*_value less than 0.1 and 0.05 is considered significant, respectively.

**Table S8. Baseline characteristics of participants for COVID-19 breakthrough infection analysis.**

|  | |  | **Young group (n=50)** | | | **Elderly group (n=98)** | | |  |
| --- | --- | --- | --- | --- | --- | --- | --- | --- | --- |
|  | **Breakthrough infection** | | **Yes (n=45)** | **No (n=5)** | ***p*** | **Yes (n=84)** | **No (n=14)** | ***p*** |  |
|  | **Age** | |  |  |  |  |  |  |  |
|  | years | | 50 (26-59) | 51 (38-59) | 0.523 | 67.5 (60-80) | 70.5 (61-79) | 0.115 |  |
|  | **Sex** | |  |  | 0.396 |  |  | 0.363 |  |
|  | Female | | 23 (51.11%) | 1 (20.00%) |  | 41 (48.81%) | 5 (35.71%) |  |  |
|  | Male | | 22 (48.89%) | 4 (80.00%) |  | 43 (51.19%) | 9 (64.29%) |  |  |
|  | **BMI** | |  |  | 1.000 |  |  | **0.035** |  |
|  | <25 (kg/m^2^) | | 27 (60.00%) | 3 (60.00%) |  | 47 (55.95%) | 12 (85.71%) |  |  |
|  | ≥25 (kg/m^2^) | | 18 (40.00%) | 2 (40.00%) |  | 37 (44.05%) | 2 (14.29%) |  |  |
|  | **Laboratory tests^a^** | |  |  |  |  |  |  |  |
|  | ALT (U/L) | | 20.19 (10.44-83.68) | 21.20 (10.26-78.55) | 0.925 | 20.72 (6.13-62.09) | 24.17 (12.66-51.47) | 0.377 |  |
|  | AST (U/L) | | 21.98 (12.23-57.31) | 19.76 (15.72-46.77) | 1.000 | 22.52 (6.02-116.40) | 22.69 (16.82-62.59) | 0.859 |  |
|  | TBIL (µmol/L) | | 15.27 (7.32-66.05) | 9.88 (8.99-14.01) | **0.016** | 14.61 (5.11-34.03) | 11.68 (8.30-22.76) | **0.049** |  |
|  | TC (mmol/L) | | 4.56 (1.86-6.46) | 5.02 (4.09-5.99) | 0.278 | 4.88 (1.70-7.61) | 4.95 (3.17-6.94) | 0.649 |  |
|  | TG (mmol/L) | | 1.43 (0.48-6.82) | 1.29 (0.68-3.17) | 0.995 | 1.76 (0.52-8.87) | 1.75 (0.83-6.06) | 0.474 |  |
|  | GLU (mmol/L) | | 5.33 (1.97-12.88) | 5.91 (4.90-6.27) | 0.694 | 6.04 (3.47-25.72) | 7.22 (5.22-13.10) | **0.018** |  |
|  | BUN (mmol/L) | | 5.35 (1.52-10.32) | 4.26 (3.75-6.33) | 0.397 | 6.08 (2.41-11.82) | 6.54 (5.16-10.74) | 0.104 |  |
|  | CRE (mmol/L) | | 68.34 (46.80-88.54) | 61.73 (50.52-66.69) | 0.059 | 69.49 (42.20-115.11) | 71.88 (52.63-103.35) | 0.972 |  |
|  | **Underlying diseases** | | 10 (22.22%) | 2 (40.00%) | 0.741 | 60 (71.43%) | 12 (85.71%) | 0.427 |  |
|  | **Type 2 diabetes** | | 1 (2.22%) | 1 (20.00%) | 0.192 | 12 (14.29%) | 6 (42.86%) | **0.029** |  |
|  | **Hypertension** | | 3 (6.67%) | 0 | 1.000 | 18 (21.43%) | 0 | 0.123 |  |
|  | **Coronary heart disease** | | 2 (4.44%) | 0 | 1.000 | 6 (7.14%) | 1 (7.14%) | 1.000 |  |
|  | **Cor pulmonale** | | 0 | 0 |  | 1 (1.19%) | 1 (7.14%) | 0.267 |  |
|  | **Emphysema** | | 0 | 0 |  | 3 (3.57%) | 0 | 1.000 |  |
|  | **Chronic bronchitis** | | 0 | 0 |  | 11 (13.10%) | 2 (14.29%) | 1.000 |  |
|  | **Fatty liver disease** | | 3 (6.67%) | 1 (20.00%) | 0.353 | 9 (10.71%) | 2 (14.29%) | 1.000 |  |
|  | **Hypothyroidism** | | 1 (2.22%) | 0 | 1.000 | 0 | 0 |  |  |
|  |  | |  |  |  |  |  |  |  |

Data are median (range) or n (%). ^a^Laboratory test indices are presented in abbreviations: ALT, alanine aminotransferase; AST, aspartate aminotransferase; TBIL, total bilirubin; TC, total cholesterol; TG, triglyceride; GLU, blood glucose; BUN, blood urea nitrogen; CRE, creatinine.
