# Supplementary material for: Safety, immunogenicity and protective effect of sequential vaccination with inactivated and recombinant protein COVID-19 vaccine in the elderly: a prospective longitudinal study
Source: Signal Transduct Target Ther. 2024 May 13;9:129. doi: 10.1038/s41392-024-01846-9 (PMC11091094; doi:10.1038/s41392-024-01846-9)
Supplement: Supplementary file 2 — Data S1 - protocol [file 41392_2024_1846_MOESM2_ESM.docx]

**Data S1**

**A prospective, multicenter controlled trial of heterologous sequential vaccination against COVID-19 in the elderly**

**Protocol**

Version 2.0 - January 26, 2022

| Sponsor: | The Fifth Medical Center of Chinese PLA General Hospital |
| --- | --- |
| Principal investigator: | Fu-Sheng Wang |
| Ethics Reference Number: | KY-2021-7-7-2 |

| ClinicalTrials.gov | NCT05012800 |
| --- | --- |

**Table of Contents**

[1. TRIAL SUMMARY 4](#_Toc162990535)

[2. INTRODUCTION 7](#_Toc162990536)

[2.1. Background 7](#_Toc162990537)

[2.2. Objectives 8](#_Toc162990538)

[2.3. Trial design 8](#_Toc162990539)

[3. METHODS 8](#_Toc162990540)

[3.1. Study setting 8](#_Toc162990541)

[3.2. Eligibility criteria 8](#_Toc162990542)

[3.2.1. Inclusion criteria 8](#_Toc162990543)

[3.2.2. Exclusion criteria 8](#_Toc162990544)

[3.3. Interventions 9](#_Toc162990545)

[3.3.1. Investigational vaccine product 9](#_Toc162990546)

[3.3.2. Intervention assignment 9](#_Toc162990547)

[3.3.3. Exit Criteria 9](#_Toc162990548)

[3.4. Outcomes 10](#_Toc162990549)

[3.4.1 Primary endpoint measures 10](#_Toc162990550)

[3.4.2 Secondary endpoint measures 10](#_Toc162990551)

[3.5. Participant timeline 11](#_Toc162990552)

[3.6. Sample size 12](#_Toc162990553)

[3.7. Recruitment 12](#_Toc162990554)

[3.8. Allocation and vaccine management 12](#_Toc162990555)

[3.8.1. Allocation 12](#_Toc162990556)

[3.8.2. Vaccine management 12](#_Toc162990557)

[3.9. Data collection and management 13](#_Toc162990558)

[3.10. Statistical consideration 13](#_Toc162990559)

[3.10.1. Analysis of population and missing data 13](#_Toc162990560)

[3.10.2. Statistical methods 14](#_Toc162990561)

[3.11. Safety/harms 17](#_Toc162990562)

[3.12. Auditing 18](#_Toc162990563)

[4. ETHICS AND DISSEMINATION 18](#_Toc162990564)

[4.1. Research ethics approval 18](#_Toc162990565)

[4.2. Protocol amendments 19](#_Toc162990566)

[4.3. Informed consent process 19](#_Toc162990567)

[4.4. Confidentiality 19](#_Toc162990568)

[4.5. Declaration of interests 19](#_Toc162990569)

[4.6. Access to data 19](#_Toc162990570)

[4.7. Ancillary and post-trial care 19](#_Toc162990571)

[4.8. Dissemination policy 19](#_Toc162990572)

[5. STUDY ADMINISTRATION 20](#_Toc162990573)

[5.1. Key contacts 20](#_Toc162990574)

[5.2. Roles and responsibilities 20](#_Toc162990575)

[5.2.1. Protocol contributors 20](#_Toc162990576)

[5.2.2. Sponsor and Funding and Collaborators 20](#_Toc162990577)

[5.2.3. Trial committees 21](#_Toc162990578)

[6. References 22](#_Toc162990579)

[7. APPENDICES 23](#_Toc162990580)

[*7.1 Revision History* 23](#_Toc162990581)

# 1. TRIAL SUMMARY

| Title | A prospective, multicenter controlled trial of heterologous sequential vaccination against COVID-19 in the elderly |
| --- | --- |
| Primary registry and trial identifying number | ClinicalTrials.gov NCT05012800 |
| Secondary identifying numbers | KY-2021-7-7-2 |
| Sources of monetary or material support | The Emergency Key Program of Guangzhou Laboratory |
| Primary sponsor | The Fifth Medical Center of Chinese PLA General Hospital |
| Central contact | Principal Investigator: Fu-Sheng Wang, MD, Ph.D.  86-10-66933332 [fswang302@163.com](mailto:fswang302@163.com)  Investigator and coordinator: Junliang Fu, MD, Ph.D.  86- 10-66933331 [fjunliang@163.com](mailto:fjunliang@163.com)  Study Sites:  Hunyuan County People's Hospital, Datong 037499, Shanxi Province, China  Contact: Hong-Hong Liu, MD, Ph.D.  The Fifth Medical Center of Chinese PLA General Hospital, Beijing 100039, China  Contact: Yunbo Xie, MD, Ph.D. |
| Study officials/Investigators | Study Principal Investigator  Fu-Sheng Wang, MD, Ph.D.  The Fifth Medical Center of Chinese PLA General Hospital |
| Brief title | Heterologous sequential vaccination against COVID-19 in the elderly |
| Countries of recruitment | P. R. China |
| Condition(s) or focus of study | COVID-19 vaccination |
| Interventions | Two doses of inactivated BBIBP-CorV vaccines and a 3^rd^ dose of recombinant protein ZF2001 vaccine booster. |
| Key eligibility criteria | **Inclusion criteria**   1. Signed informed consent; 2. In females, the urine pregnancy test was negative; 3. At least 6 months of follow-up was completed; 4. HBsAg, anti-HCV, HIV, treponema pallidum hemagglutination assay (TPHA) screening was negative; 5. Armpit temperature was ≤37.0℃; 6. The young group was aged 18–59 years, and the elderly group between 60–80 years.   **Exclusion criteria**   1. In females, the urine pregnancy test positive; 2. Pregnant or lactating women; 3. Known allergies to certain components of these two vaccines; 4. Patients with serious chronic diseases or advanced diseases such as high blood pressure, diabetes, asthma, thyroid disease, *etc*. which cannot be controlled by drugs; 5. Those suffering from thrombocytopenia, hemorrhagic disease or thrombotic disease; 6. Those with congenital or acquired angioedema/neuroedema; 7. Those with a history or family history of convulsions, epilepsy, encephalopathy, other progressive neurological diseases, and psychiatric disorders; 8. Lymphadenopathy； 9. Lymphoma, leukemia and other systemic malignancies; 10. Autoimmune disease; 11. Chronic diseases with cute exacerbation or acute infectious diseases and fever. |
| Study design | Study type: Prospective real-world interventional trial  Allocation: Open, non-randomized  Intervention model: Parallel group  Primary purpose: Prevention |
| Date of enrolment | July 1, 2021 |
| Target sample size | 300 (200 in the elderly group, 100 in the young group) |
| Recruitment status | Recruited |
| Primary outcomes | **Primary endpoint measures**   - Safety profiles within one month after each vaccination dose, including:   1. The incidence of adverse events;   2. Liver function;   3. Kidney function;   4. Blood glucose;   5. Blood lipids. - The definition and grade of adverse events (AEs) were evaluated according to the Common Terminology Criteria for Adverse Events (CTCAE), version 5.0:  1. Grade 1 (mild) - asymptomatic or mild symptoms, clinical or diagnostic observations only, intervention not indicated; 2. Grade 2 (moderate) - minimal, local, or non-invasive intervention indicated, limiting age-appropriate instrumental activities of daily living (ADL); 3. Grade 3 (severe or medically significant but not immediately life-threatening) - indication for hospitalization or prolongation of hospitalization, disabling and limiting self-care ADL. |
| Secondary outcomes | **Secondary endpoint measures**   - Immune protection of the participants:   - - The titers of neutralizing and anti-receptor binding domain (RBD) antibodies at the baseline and the 1^st^, 2^nd^, 4^th^, 7^th^, 8^th^, 10^th^, 13^th^, and 16^th^ months after the first vaccination in each study group. |

# 2. INTRODUCTION

## 2.1. Background

Coronavirus disease 2019 (COVID-19), an emergent acute respiratory infectious disease, has caused unprecedented morbidity and mortality during the pandemic^[1-2]^. Current drug treatment options are limited, making the establishment of an immune barrier through safe and effective vaccination a crucial strategy to control the virus’s spread^[3-4]^. The focus of vaccine research and development is on preventing antibody-mediated infection enhancement and vaccine-enhanced disease. Globally, 83 vaccines are undergoing clinical trials, with 184 vaccine candidates in preclinical evaluation^[5]^. Five technical routes for vaccine development are being pursued in China: inactivated, adenovirus vectors, recombinant subunits, nucleic acids, and attenuated influenza virus vectors. Five novel coronavirus vaccines have been conditionally marketed for emergency use^[6]^. Considering the “safety of the vaccine itself,” the Chinese Center for Disease Control and Prevention’s Novel Coronavirus Vaccine Working Group has drafted “Technical Guidelines for Novel Coronavirus Vaccination,” which outlines 31 conditions for vaccination. According to the National Health Commission, as of June 29, 2022, China has received over 1.2 billion doses of the COVID-19 vaccine, aiming to vaccinate at least 70% of the target population by the end of 2022, gradually establishing a herd immunity barrier.

Clinical research has demonstrated that the severity of COVID-19 is largely determined by the age of the patient, with individuals aged 65 years or older constituting 80% of hospitalizations and facing a 23-fold higher risk of death compared to those under 65^[7]^. This indicates that the elderly are at a high risk of severe disease following COVID-19 infection and have a poor clinical prognosis^[7]^. Considering the current demographic trends in China, priority should be given to vaccinating the elderly to protect them and reduce the risk of COVID-19 infection. However, the majority of available vaccine data are derived from studies involving young and middle-aged healthy individuals, with many phase II COVID-19 vaccine trials seeing limited participation from the elderly^[8-10]^. Although phase III trials consciously enrolled the elderly, individuals with comorbidities and frailty were excluded, resulting in insufficient research data on COVID-19 vaccination in the elderly^[11-12]^. In this context, this study focuses on investigating the safety and effectiveness of COVID-19 vaccination in the elderly, as well as its potential immune mechanisms. The research findings aim to provide theoretical support for the establishment of expert consensus on COVID-19 vaccination of the elderly in China.

## 2.2. Objectives

The aim of this study is to evaluate the safety and immunogenicity of the COVID-19 vaccine in the elderly aged 60–80 years.

## 2.3. Trial design

This trial is investigator-initiated and adheres to the principles of good clinical practice. It is a multi-center, prospective, single-arm clinical study. The trial will include two participant groups: an elderly group aged 60 to 80 years and a younger control group aged 18 to 59 years.

# 3. METHODS

## 3.1. Study setting

This trial will be conducted in two hospitals: the Hunyuan County People's Hospital, Datong 037499, Shanxi Province, China; and the Fifth Medical Center of Chinese PLA General Hospital, Beijing 100039, China.

## 3.2. Eligibility criteria

### 3.2.1. Inclusion criteria

1. Signed informed consent;
2. In females, the urine pregnancy test was negative;
3. At least 6 months of follow-up was completed;
4. HBsAg, anti-HCV, HIV, treponema pallidum hemagglutination assay (TPHA) screening was negative;
5. Armpit temperature was ≤37.0℃;
6. The young group was aged 18–59 years, and the elderly group between 60–80 years.

### 3.2.2. Exclusion criteria

1. In females, the urine pregnancy test positive;
2. Pregnant or lactating women;
3. Known allergies to certain components of these two vaccines;
4. Patients with serious chronic diseases or advanced diseases such as high blood pressure, diabetes, asthma, thyroid disease, etc. which cannot be controlled by drugs;
5. Those suffering from thrombocytopenia, hemorrhagic disease or thrombotic disease;
6. Those with congenital or acquired angioedema/neuroedema;
7. Those with a history or family history of convulsions, epilepsy, encephalopathy, other progressive neurological diseases, and psychiatric disorders;
8. Lymphadenopathy；
9. Lymphoma, leukemia and other systemic malignancies;
10. Autoimmune disease;
11. Chronic diseases with cute exacerbation or acute infectious diseases and fever.

## 3.3. Interventions

### 3.3.1. Investigational vaccine product

In this study, the first two doses were China's Sinopharm COVID-19 inactivated vaccine BBIBP-CorV (Vero cells) containing 4 μg per 0.5 mL in a vial. The third booster dose was Zhifei Longcom recombinant COVID-19 vaccine ZF2001 (CHO cells) containing 25 μg per 0.5 mL in a vial. Both vaccines were adjuvanted by aluminum hydroxide. All the participants received the vaccine intramuscularly through a deltoid.

### 3.3.2. Intervention assignment

Both the experimental and control groups will receive two doses of the inactivated COVID-19 vaccine, administered on day 0 (baseline) and day 25±3. A third booster dose will be given 6 months after the second dose.

### 3.3.3. Exit Criteria

The participants may exit the study for any of the following reasons:

1. Voluntarily withdrawal (due to withdrawal of informed consent, loss of informed consent capacity, lack of efficacy, intolerance, adverse events [AEs], etc.);
2. Poor compliance, inability to receive the vaccine as scheduled, or complete the study protocol;
3. Becoming pregnant or lactating during the treatment period;
4. Experiencing complications, AEs, serious AEs (SAEs), or special physiological changes occurring during vaccination that lead the investigator to judge further vaccination as unsuitable;
5. Any other condition deemed by the investigator as necessitating withdrawal from the study.

## 3.4. Outcomes

### 3.4.1 Primary endpoint measures

- Safety profiles within one month after each vaccination dose, including:

1. The incidence of adverse events;
2. Liver function;
3. Kidney function;
4. Blood glucose;
5. Blood lipids;

- The definition and grade of AEs were evaluated according to the Common Terminology Criteria for Adverse Events, version 5.0:

1. Grade 1 (mild) - asymptomatic or mild symptoms, clinical or diagnostic observations only, intervention not indicated;
2. Grade 2 (moderate) - minimal, local, or non-invasive intervention indicated, limiting age-appropriate instrumental activities of daily living (ADL);
3. Grade 3 (severe or medically significant but not immediately life-threatening) - indication for hospitalization or prolongation of hospitalization, disabling and limiting self-care ADL.

### 3.4.2 Secondary endpoint measures

- Immune protection of the participants:
  - - The titers of neutralizing and anti-receptor binding domain (RBD) antibodies at the baseline and the 1^st^, 2^nd^, 4^th^, 7^th^, 8^th^, 10^th^, 13^th^, and 16^th^ months after the first vaccination in each study group.

## 3.5. Participant timeline

Figure 1. The schedule of enrolment, interventions, and assessments.

| **Visits** | **Screening** | **V1** | **V2** | **V3** | **V4** | **V5** | **V6** | **V7** | **V8** | **V9** |
| --- | --- | --- | --- | --- | --- | --- | --- | --- | --- | --- |
| **Timepoint (month)** | **Day**  **-21~0** | **Day 0** | **1^st^** | **2^nd^** | **4^th^** | **7^th^** | **8^th^** | **10^th^** | **13^th^** | **16^th^** |
| **Time window (day)** |  | **± 3** | **± 3** | **± 15** | **± 15** | **± 3** | **± 15** | **± 15** | **± 15** | **± 15** |
| **ENROLMENT:** | | | | | | | | | | |
| Informed consent | × |  |  |  |  |  |  |  |  |  |
| Eligibility screen | × |  |  |  |  |  |  |  |  |  |
| Demographical characteristics | × |  |  |  |  |  |  |  |  |  |
| Medical history | × |  |  |  |  |  |  |  |  |  |
| Urine pregnancy test  (reproductive women) | × |  | × | × | × | × | × | × | × | × |
| Height, weight, blood pressure | × |  |  |  |  |  |  |  |  |  |
| Underarm temperature | × |  |  |  |  |  |  |  |  |  |
| Lab tests & Images*^#^* | × |  |  |  |  |  |  |  |  |  |
| **INTERVENTIONS:** | | | | | | | | | | |
| Allocation |  | × |  |  |  |  |  |  |  |  |
| Vaccination |  | × | × |  |  | × |  |  |  |  |
| 30 minutes’ observing |  | × | × |  |  | × |  |  |  |  |
| **ASSESSMENTS:** | | | | | | | | | | |
| Lab tests*** | × |  | × | × | × | × | × | × | × | × |
| Distribute/recycle diary cards |  | × | × | × | × | × | × | × | × | × |
| Safety (AEs) |  | × | × | × | × | × | × | × | × | × |
| Safety (SAEs) |  | × | × | × | × | × | × | × | × | × |
| Concomitant care |  | × | × | × | × | × | × | × | × | × |
| Humoral immunoassays |  | × | × | × | × | × | × | × | × | × |

# Including hepatitis B serologic, alpha-fetoprotein (AFP), carcinoembryonic antigen (CEA), Anti-HCV, HIV antibody, TPHA, electrocardiogram (ECG), chest x-ray, abdominal ultrasound (liver, gallbladder, spleen, pancreas, kidney). * Including liver and kidney function, blood glucose, and blood lipids.

## 3.6. Sample size

The target sample size is 300 participants. The allocation ratio will be 2:1, with 200 participants in the elderly experimental group (aged 60–80) and 100 participants in the younger control group (aged 18–59).

## 3.7. Recruitment

Participants will be recruited exclusively from two designated COVID-19 vaccination hospitals: the Fifth Medical Center of Chinese PLA General Hospital, Beijing; and the Hunyuan County People's Hospital, Shanxi Province. No other recruitment strategy (*e.g.*, advertisement) will be adopted in this study.

## 3.8. Allocation and vaccine management

### 3.8.1. Allocation

The participants will be assigned to either the experimental or control group in a 2:1 ratio based on age.

### 3.8.2. Vaccine management

**Storage conditions:** All study vaccines must be stored securely, following their specific storage requirements before distribution. Each clinical research unit will designate a specific person responsible for vaccine storage and distribution and will keep detailed records. Expired vaccines are not to be used, and attention must be paid to risks such as fire, theft, and damage from heat, mildew, insects, or rodents.

**Notes:** Vaccines should always remain in their original packaging until use. Before administering a vaccine, one should check that the packaging is intact; the vaccine should not be used if the seal is damaged or missing. All unused vaccines and their packaging should be saved.

**Vaccine inventory**: It is the investigator’s responsibility to ensure that vaccine quantities align with those registered and to maintain and record vaccine registration. In accordance with regulatory requirements, the investigator or designee must keep records of vaccine delivery throughout the entire research process, including records of the amount of research vaccine received, stored, and/or distributed, and the amount of vaccine returned by the participant to the research center.

## 3.9. Data collection and management

The data for this study will be recorded and managed through an electronic data capture system. Before locking the database, data managers will review and discuss the data with principal investigators and clinical research partners and then produce a data management report. This scheme provides only general requirements for data management.

1. Completed case report forms are reviewed by clinical investigators and supervisors and subsequently submitted to the data statistics unit for data entry and management. Documentation of all processes is required.
2. Data entry and management are the responsibility of the data administrator of the statistical unit. The electronic database will be used for these purposes. To ensure data accuracy, double-entry and proofreading are to be carried out independently by two data administrators. If there are queries in the case report form (CRF), the data manager will fill in a question form and send it to the investigator via the supervisors. The investigator is expected to respond promptly. The data manager will modify, confirm, and enter the data according to the investigator’s answer and may resend the query form if necessary.
3. After data review and database confirmation, the database will be locked by the principal investigator, clinical research partners, and statistical analysts.
4. Next, the statistical plan will be discussed by the principal investigator, statistician, and clinical research partner. Modifications to the database after locking can only be made with the written consent of the clinical research leader, clinical research partner, statistician, or data manager.
5. After the database is locked, the data manager will import it into the designated database and submit it to the statistician for statistical analysis. The statistician will subsequently write the statistical analysis report.

## 3.10. Statistical consideration

### 3.10.1. Analysis of population and missing data

**Full Analysis Set (FAS):** According to the intention-to-treat (ITT) principle, this study aims to include as many participants as possible. Participants who did not complete all three doses of vaccination will be excluded.

**Per-Protocol Set (PPS):** Based on the FAS population, participants with poor compliance will be excluded. Specifically, this set excludes individuals who participated in follow-up no more than six times.

**Safety Set (SS):** Participants with at least one vaccination record and measured AEs/SAEs correspondingly.

**Missing Data Processing:** The missing data on demographic information, safety indicators, and immunogenicity indicators for the FAS and PPS populations will not be imputed.

### 3.10.2. Statistical methods

**Principles of statistical analysis:**

All statistical analyses were performed using IBM SPSS (version 26).

Demographic analysis will be performed in FAS population, the primary analysis (safety) will be performed in the SS population, and the secondary analysis (immunogenicity) will be performed both in the FAS and PPS population.

The Kolmogorov-Smirnov test was used to evaluate the distribution types. Non-normally distributed and ordered data are represented by the median (range). Measurement of pseudovirus 50% neutralization titers (pVNT_50_s) are represented by geometric means. Pearson’s chi-square test, continuity correction, and Fisher’s exact test were used to check the proportion of the count data.

The Mann-Whitney U test and paired t-test were used to compare differences between groups, the Wilcoxon rank-sum test was used to compare intragroup differences, and the nest t-test was used to compare unmatched groups. We displayed the median values of each group in the figures using GraphPad Prism 8 and estimated the differences based on the 95% confidence interval (CI).

Baseline characteristics were screened using univariate logistic regression, and the significantly different factors (*P*_value<0.1) were further analyzed using multivariate logistic regression. The analysis results are presented using an OR combined with a 95% CI (lower–upper) in the table.

In correlation analysis, the Pearson correlation test was used to process normally distributed datasets, while the Spearman rank correlation test was used to process non-normally distributed datasets. An absolute R value>0.5 indicated a strong correlation, whereas an absolute R value≤0.5 indicated a weak correlation.

The hypothesis test was bilateral, and a *P*_value<0.05 was considered statistically significant.

**Demographic analysis:**

The analysis of baseline demographic information will be based on the FAS population.

This includes a statistical description and comparison of baseline demographic information (including age, gender, BMI, laboratory test indices, and comorbidities) to measure comparability between the two groups. Summarized statistical data will be used based on the properties of the variables (continuous or categorical).

**Primary analysis:**

The main endpoint of this study is the safety after vaccination, which involves the evaluation of AEs, SAEs, and laboratory test indices, and performed on the SS.

The AEs/SAEs occurring in the two groups within one month after each dose of vaccination will be statistically described as a categorical variable, and Pearson’s chi-square test or Fisher’s exact test will be used for intergroup comparisons. The AEs/SAEs of the different categories will be summarized in percentages. The laboratory test indices at each follow-up point will be statistically described as continuous variables, and the Mann–Whitney U test will be used for intergroup comparisons. The median values of the laboratory test indices for the two groups will be calculated at each follow-up point, as well as the corresponding intergroup differences and 95% CIs.

**Secondary analysis:**

The secondary endpoint of this study is the immunogenicity after vaccination, which is performed on the FAS and PPS.

The antibody-positive rates at each follow-up point in the two groups will be statistically described as categorical variables, and Pearson’s chi-square test or Fisher’s exact test will be used for intergroup comparisons. The antibody titers at each follow-up point in the two groups will be statistically described as continuous variables, and the Mann–Whitney U test will be employed for intergroup comparison. For the absolute titers of neutralizing antibodies and anti-RBD antibodies, the median values of antibody titers will be calculated for the two groups at each follow-up point, as well as the corresponding intergroup differences and 95% CIs. For the relative titer values of variant-specific neutralizing antibodies, the geometric mean of the antibody titers for the two groups will be determined at each follow-up point, as well as the corresponding intergroup differences and 95% CIs.

**Breakthrough infection analysis:**

The population for breakthrough infection analysis will be based on the FAS population and exclude participants whose SARS-CoV-2 infection status during the end of the 2022 pandemic cannot be confirmed through follow-up. The **Demographic analysis** will be performed as above.

(1) Proportion of different breakthrough infection states

Based on follow-up data, the status of breakthrough infections among the participants will be statistically described, and the infection rate will be used as an intergroup categorical variable for comparison using Pearson’s chi-square test.

(2) Proportion of different types of breakthrough infection symptoms

The symptoms of symptomatic infected individuals will be statistically described and displayed as intergroup categorical variables.

(3) Duration of breakthrough infection symptoms (days)

The duration of symptoms will be statistically described using the duration as the classification criterion (if longer than 7 days) and compared as an intergroup classification variable.

(4) Correlation between infection and antibody titers

The infection status within the two groups will be statistically described, and the corresponding antibody titers will be presented as continuous variables. An intragroup comparison will be performed using the Mann–Whitney U test, and the corresponding medians and 95% CIs will be calculated.

(5) Correlation between symptom severity and antibody titers

Based on the classification criteria of whether the duration of symptoms is longer than 7 days, whether there is accompanying fever, and whether there are multisystemic symptoms, the infection symptoms of the two groups will be statistically described. The corresponding antibody titers will be considered continuous variables; the Mann–Whitney U test will be employed for the intragroup comparison, and the corresponding medians and 95% CIs will be determined .

**Pseudovirus neutralization test analysis**

Based on the FAS population, an equal number of participants were randomly selected from both groups, ensuring no significant intergroup differences in gender and age, and no missing values at baseline and follow-up points at the 2^nd^, 7^th^, 8^th^, 13^th^, and 16^th^ months. The analysis of antibody-positive rates and titers (pVNT_50_s) is in the same way as the **Secondary analysis** mentioned above.

(1) Correlation between pVNT_50_s and symptoms

The infection status within the two groups will be statistically described, and the corresponding pVNT_50_s will be treated as continuous variables. An intergroup comparison will be performed using the nested t-test, and the corresponding geometric means and 95% CIs will be provided.

(2) Correlations between wild-type SARS-CoV-2–specific pVNT_50_s and variant-specific pVNT_50_s

The pVNT_50_s measured at each follow-up point will be statistically described as continuous variables. The Kolmogorov–Smirnov test will be utilized to evaluate data normality, followed by the Pearson correlation test or the Spearman rank correlation test for correlation analysis.

(3) Correlations between different variant-specific pVNT_50_s

The pVNT_50_s measured at each follow-up point will be statistically described as continuous variables. The Kolmogorov–Smirnov test will be employed to evaluate data normality, followed by the Pearson correlation test or the Spearman rank correlation test for correlation analysis.

## 3.11. Safety/harms

AEs and SAEs will be monitored and recorded from the time the participant signed the informed consent form to the completion of the follow-ups. AEs/SAEs will be recorded in detail, including onset date, duration, severity, treatment, and relation to the investigational medical product. All AEs/SAEs will be followed up until finalized (recovery/relief, stability, death, or other explainable circumstances, such as lost to follow-up).

Once identified, SAEs must be acted upon and reported within 24 hours. They will be reported to the Institutional Review Board (IRB) as soon as possible.

## 3.12. Auditing

1. All research centers participating in clinical trials should adopt standard operating procedures to ensure the implementation of quality control and quality assurance systems for clinical trials;
2. To ensure the quality of the multi-center trial, before starting the formal trial, the principal investigators of each study site shall jointly discuss and formulate the protocol, and the relevant investigators shall be trained at the same time;
3. Each study site shall implement the trial according to a unified protocol;
4. Each study site will implement the same procedure to manage the trial vaccines, and the experimental vaccine manager must carefully store vaccines in accordance with the storage conditions proposed by the unit leader.
5. All observed results and abnormal findings in the clinical trials should be carefully verified and recorded in a timely manner to ensure the reliability of the data. All kinds of instruments, equipment, reagents, and standards used in the clinical trials must meet strict quality standards and function correctly under normal conditions. The recording and transfer of clinical data must be carried out by experienced investigators and supervised or verified to ensure data accuracy and scientific integrity. Clinical trial conclusions must be derived from the raw data.
6. The investigators in charge of the study should fill in the research medical records and CRF on time, completely, in detail, and accurately and submit or store documents according to the prescribed procedures after being signed and confirmed by a superior; all data related to the study must be centrally managed and analyzed.
7. To ensure the reliability and completeness of the trial data, the principal investigators of each study site, clinical research partners, and supervisors will monitor their clinical systems regularly to judge whether the implementation of the trial is consistent with the protocol and whether the reported data are consistent with the records of the study sites. A report will follow every visit.

# 4. ETHICS AND DISSEMINATION

## 4.1. Research ethics approval

The protocol and informed consent forms for this trial have been approved by the institutional review boards of the Fifth Medical Center of the Chinese PLA General Hospital. Major protocol amendments and suggestions for managing SAEs will also be reported to the IRB.

## 4.2. Protocol amendments

Any major changes, such as alterations to study objectives, study design, participant population, sample sizes, study procedures, or outcomes that might impact the potential benefit or risk to participants, will require a formal amendment to the protocol and approval from the IRB.

## 4.3. Informed consent process

Before beginning the trial, the investigators must clearly explain the informed consent form to each participant and obtain their voluntary signed informed consent.

## 4.4. Confidentiality

The investigators are obligated to maintain the confidentiality of all participant records that contain names or other personal identifiers. All research data will be identified by a study participant ID only. Data or records shall not be used for purposes other than this clinical study.

## 4.5. Declaration of interests

The principal investigators declare that they have no financial or other competing interests.

## 4.6. Access to data

All investigators who are members of the steering committee will be given full access to the final data sets.

## 4.7. Ancillary and post-trial care

The investigators will continue to follow up with participants until the 19^th^ month for safety and research purposes. Long-term data regarding efficacy and safety will be collected.

## 4.8. Dissemination policy

The trial results will be communicated to health authorities, professionals, and participants involved in this study. The results of this trial will be published as soon as they become available. Further publications and authorship related to this study’s results must be reviewed by the principal investigator and sponsor, and written consent must be obtained. The data sharing policy will be described in detail in the data sharing statement upon the publication of the study’s results.

# 5. STUDY ADMINISTRATION

## 5.1. Key contacts

**Central contact**

Principal Investigator: Fu-Sheng Wang, MD, Ph.D. 86-10-66933332 [fswang302@163.com](mailto:fswang302@163.com)

Investigator and coordinator: Junliang Fu, MD, Ph.D. 86-10-66933331 [fjunliang@163.com](mailto:fjunliang@163.com)

Study Sites:

Hunyuan County People's Hospital, Shanxi, China, 037400

Contact: Hong-Hong Liu, MD, Ph.D.

The Fifth Medical Center of Chinese PLA General Hospital, Beijing, China, 100039

Contact: Yun-Bo Xie, MD, Ph.D.

## 5.2. Roles and responsibilities

### 5.2.1. Protocol contributors

Fu-Sheng Wang, Junliang Fu, and Peng-Hui Yang designed the trial.

Bao-Peng Yang developed a data management and statistical plan.

Hong-Hong Liu and Yunbo Xie drafted the protocol.

### 5.2.2. Sponsor and Funding and Collaborators

**Sponsor**

The Fifth Medical Center of Chinese PLA General Hospital.

**Funding**

The Emergency Key Program of Guangzhou Laboratory.

**Collaborators**

***Study sites:***

Hunyuan County People's Hospital of Shanxi Province.

***Vaccine provider:***

Beijing Institute of Biological Products Co., Ltd. of Sinopharm CNBG produces inactivated COVID-19 vaccine BBIBP-CorV (Vero cells);

Anhui Zhifei Longcom Biopharmaceutical Co., Ltd. produces recombinant COVID-19 vaccine ZF2001 (CHO cells).

***Electronic source data system and eCRF system provider:***

Beijing AIGenius Data Technology Co., Ltd.

***Trial monitoring/Data management team/Clinical research organization:***

SciTrials Medical Development Co., Ltd.

**Statistical analysis**

The Fifth Medical Center of Chinese PLA General Hospital.

### 5.2.3. Trial committees

**Steering Committee:** Fu-Sheng Wang, Junliang Fu, Peng-Hui Yang.

**Operating Committee:** Hong-Hong Liu, Yunbo Xie, Bao-Peng Yang, Huan-Yue Wen, Jin-E Lu, Yan Liu, Xi Chen, Meng-Meng Qu, Yang Zhang, Wei-Guo Hong, Yong-Gang Li.

# 6. References

1. Meo, S. A. et al. Novel coronavirus 2019-nCoV: prevalence, biological and clinical characteristics comparison with SARS-CoV and MERS-CoV. *Eur. Rev. Med. Pharmacol. Sci.* **24**, 2012-2019 (2020).
2. Meo, S. A. et al. Biological and epidemiological trends in the prevalence and mortality due to outbreaks of novel coronavirus COVID-19. *J. King Saud. Univ. Sci.* **32**, 2495-2499 (2020).
3. Dong, Y. et al. A systematic review of SARS-CoV-2 vaccine candidates. *Signal Transduct. Target. Ther.* **5**, 237 (2020).
4. Krause, P. R. & Gruber, M. F. Emergency use authorization of COVID vaccines—safety and efficacy follow-up considerations. *N. Engl. J. Med.* **383**, e107 (2020).
5. Singh, J. A. & Upshur, R. E. G. The granting of emergency use designation to COVID-19 candidate vaccines: implications for COVID-19 vaccine trials. *Lancet Infect. Dis.* **21**, e103–09 (2021).
6. Zhu, N. et al. A novel coronavirus from patients with pneumonia in China, 2019. *N. Engl. J. Med.* **382**, 727–33 (2020).
7. De Salazar, P. M. et al. Lockdown measures and relative changes in the age-specific incidence of SARS-CoV-2 in Spain. *Epidemiol. Infect.* **148**, e268 (2020).
8. Dal-Ré, R. Orenstein, W. & Caplan, A. L. Trial participants’ rights after authorization of COVID-19 vaccines. *Lancet Respir. Med.* 9, e30–31 (2021).
9. Xia, S. et al. Effect of an inactivated vaccine against SARS-CoV-2 on safety and immunogenicity outcomes: interim analysis of 2 randomized clinical trials. *JAMA* **324**, 951-960 (2020).
10. Baden, L.R. et al. Efficacy and safety of the mRNA-1273 SARS-CoV-2 vaccine. *N. Engl. J. Med.* **384**, 403-416 (2021).
11. Mueller, A. L. McNamara, M. S. & Sinclair, D. A. Why does COVID-19 disproportionately affect older people? *Aging (Albany NY)* **12**, 9959–9981 (2020).
12. Westemeier, C. L. et al. Impaired cytotoxic CD8 T cell response in elderly COVID-19 patients. *mBio* **11**, e02243–e20 (2020).

# 7. APPENDICES

## *7.1 Revision History*

| **Version** | **Date** | **Amendment Text** | **Description** |
| --- | --- | --- | --- |
| 1.1 | March 6, 2020 | **Vaccination plan adjustment:**  The original statement “the participants will complete two doses of inactivated COVID-19 vaccine on day 0 and day 25±3” was modified to “the participants will receive a third dose of recombinant protein subunit vaccine 6 months after completing their two-dose regimen of inactivated COVID-19 vaccine.” | Due to the current pandemic, the national public health policy now mandates the administration of a third booster dose for vaccination. |
|  |  | **Follow-up time adjustment:**  Follow-up assessments are scheduled at the 1^st^, 3^rd^, 6^th^, and 9^th^ months following the administration of the two vaccine doses. The timing was adjusted to the 1^st^, 2^nd^, 4^th^, 7^th^, 8^th^, 10^th^, 13^th^, and 16^th^ months after the initial vaccine dose. | We need to conduct follow-up assessments in the 1^st^, 3^rd^, 6^th^, and 9^th^ months following the final vaccination to assess its safety and immune protection. |
| 2.0 (Final) | January 26, 2022 | **Items of laboratory test adjustment:**  Blood routine, liver and kidney function, blood glucose, blood lipids, and coagulation function will be tested at each follow-up point, adjusted to only test liver and kidney function, blood sugar, and blood lipids starting from the 7^th^ month (3^rd^ dose of vaccination). | The primary objective of performing blood routine and coagulation function tests is to assess safety post-vaccination. Following the initial two vaccine doses, no adverse reactions associated with coagulation or blood count were observed among the participants. The majority of the project participants are elderly individuals who are resistant to frequent and extensive blood collection, consequently leading to a reduced willingness for follow-up. To ensure compliance with follow-up procedures, the blood routine and coagulation function tests were omitted without compromising the scientific integrity of the project. |
